# Assessment of the Fairness of Privacy Policies of Mobile Health Apps: Scale Development and Evaluation in Cancer Apps

**DOI:** 10.2196/17134

**Published:** 2020-07-28

**Authors:** Jaime Benjumea, Jorge Ropero, Octavio Rivera-Romero, Enrique Dorronzoro-Zubiete, Alejandro Carrasco

**Affiliations:** 1 Department of Electronic Technology Universidad de Sevilla Sevilla Spain

**Keywords:** privacy, mhealth apps, fairness assessment scale, cancer apps, GDPR

## Abstract

**Background:**

Cancer patients are increasingly using mobile health (mHealth) apps to take control of their health. Many studies have explored their efficiency, content, usability, and adherence; however, these apps have created a new set of privacy challenges, as they store personal and sensitive data.

**Objective:**

The purpose of this study was to refine and evaluate a scale based on the General Data Protection Regulation and assess the fairness of privacy policies of mHealth apps.

**Methods:**

Based on the experience gained from our previous work, we redefined some of the items and scores of our privacy scale. Using the new version of our scale, we conducted a case study in which we analyzed the privacy policies of cancer Android apps. A systematic search of cancer mobile apps was performed in the Spanish version of the Google Play website.

**Results:**

The redefinition of certain items reduced discrepancies between reviewers. Thus, use of the scale was made easier, not only for the reviewers but also for any other potential users of our scale. Assessment of the privacy policies revealed that 29% (9/31) of the apps included in the study did not have a privacy policy, 32% (10/31) had a score over 50 out of a maximum of 100 points, and 39% (12/31) scored fewer than 50 points.

**Conclusions:**

In this paper, we present a scale for the assessment of mHealth apps that is an improved version of our previous scale with adjusted scores. The results showed a lack of fairness in the mHealth app privacy policies that we examined, and the scale provides developers with a tool to evaluate their privacy policies.

## Introduction

### Privacy in Mobile Health Apps

Health care systems are putting a great emphasis on the role of the patient and encouraging people to take control of their health [1]. Mobile health (mHealth) apps are one of the technological breakthroughs that make this possible. There are more than 3 billion smartphone users worldwide, and this number is predicted to grow by several 100 million in the next few years [2]. This proliferation of smartphones has led to an increase in the availability and abundance of mHealth apps. In 2017, there were more than 300,000 mHealth apps, and this number tends to grow by 25% every year. In 2018, 52% of smartphone users collected health-related information on their smartphones, and 60% of smartphone users downloaded health-related apps [3].

Among other uses, mHealth apps can provide disease and treatment information; practical tools for avoiding some diseases (prevention and healthy behavior promotion); tools to assist in the identification of symptoms (early detection); practical tools to deal with the medical, behavioral, or emotional aspects of a specific disease (disease management); and access to peer or professional assistance (support) [4,5].

Despite the potential impact of mHealth apps on patient health, there is a lack of specific regulations and standards regarding the development of mHealth apps [6], which may result in potential risks and poor mHealth app quality. For example, some studies have reported problems with existing mHealth apps such as failure to meet the needs of persons with chronic conditions [7], a lack of cited source material or references [5], and insufficient testing of mHealth apps with respect to usability and validity [8]. Such setbacks reduce health care professional and patient confidence in these apps [9,10].

Criteria have been proposed to assess mHealth apps. Stoyanov et al [11] developed the Mobile Application Rating Scale (MARS) scale to classify and rate the quality of mHealth apps based on a literature review of app evaluations containing explicit quality rating criteria. Llorens-Vernet and Miró [6] recently proposed criteria to be integrated into a general standard for mHealth app development based on a systematic review, searches on professional organization websites, and standards governing the development of software for medical devices.

Privacy is a major concern for mHealth app users [12], as some mHealth apps require the collection, storage, and sharing of personal and sensitive patient data. Guidelines and recommendations for health-related apps—such as those developed by the Andalusian Agency for Healthcare Quality (Spain), Tecnologies de la Informació i la Comunicació Salut Social Foundation (Spain), National Health Service (United Kingdom), and European Commission—include several privacy items that highlight its importance in the context of mHealth. Also, privacy is one of the components included in the criteria proposed by Stoyanov et al [11] and by Llorens-Vernet and Miró [6].

Privacy assessment is a multifaceted issue that, among other things, bears upon privacy policies. In the context of mHealth apps, privacy policies contain privacy-related information that users can review prior to installation in order to get a clear idea of what personal data the app will access and the purposes for their processing. These concerns should be taken seriously, but 70% of the 600 most-used health-related apps do not include a privacy policy [13]. Although the previously mentioned guidelines and recommendations, MARS scale, and criteria include items regarding privacy, developers and evaluators need more specialized tools when it comes to the development and assessment of mHealth app privacy policies.

In this paper, we introduce a novel scale based on the General Data Protection Regulation (GDPR) to assess the fairness of mHealth app privacy policies. This scale provides detailed information regarding items to be included in privacy policies in order to comply with the GDPR. Consequently, we offer an objective and reproducible method for assessing the fairness of mHealth app privacy policies or developing the privacy policy of an mHealth app. This paper presents the final version of the scale (the culmination of an iterative development process) and the results obtained after applying it in a case study.

### Legal Background

The GDPR is a regulation (2016/79) passed by the European Parliament and the Council of the European Union (EU). It was published in the Official Journal of the European Union [14] in 2016 and has been applicable since May 25, 2018. The GDPR applies to all EU member countries plus Iceland, Luxemburg, and Norway. Being a regulation (and not a directive), the GDPR applies directly to all these countries.

The GDPR introduces some important changes that replace previous legislation (Directive 95/46/EC). The first one can be found in Article 3 (territorial scope), as the GDPR applies to any controller or processor in the EU, even if processing does not take place in the EU. Also, the GDPR applies to any controller or processor (regardless the country of origin) if it is related to “the offering of goods or services, irrespective of whether a payment of the data subject is required, to such data subjects in the Union or the monitoring of their behavior as far as their behavior takes place within the Union” (Article 3.2). The controller must be able to demonstrate compliance with the GDPR (Article 5.2) and is subject to higher fines than before (Articles 66 and 83). Article 4 of the GDPR includes definitions that clarify relevant concepts. These concepts are summarized in Table 1.

Like many legal texts, the GDPR is difficult to understand and comply with, especially when it comes to app developers or users who are not legal experts; reading, interpreting, and understanding 99 articles in 88 pages of legal language is not easy. Our paper helps ordinary people become more familiar with the regulations so they can comply with the laws. We have developed a tool that makes compliance as easy as following simple guidelines such that even small app developers (eg, freelancers) can easily use it.

For example, there are two items in our scale (items 4 and 5) regarding the information to be provided to the data subject, described in Article 13 with a simple sentence: “the purposes of the processing for which the personal data are intended as well as the legal basis for the processing.” This sentence is translated in the definition of our scale into approximately 250 words because this article is connected with several parts of the GDPR: recitals 39, 58, 60, 61, and 63, and articles 4, 5, and 6. These recitals and articles must be read and understood to be clear on the intentions of the GDPR.

**Table 1:** Definition of General Data Protection Regulation concepts.

Concept	Definition
Data subject	A natural person whose personal data are being processed; the GDPR^a^ defines personal data not only as the data related to an identified person, but also as the data that can be used to identify, directly or indirectly, a natural person.
Data controller	“The natural or legal person, public authority, agency, or other body which, alone or jointly with others, determines the purposes and means of the processing of personal data” [14].
Data processor	“The natural or legal person, public authority, agency, or other body which processes personal data on behalf of the controller” [14].
Recipient	“The natural or legal person, public authority, agency, or another body, to which the personal data are disclosed” [14].
Representative	A natural or legal person established in the EU^b^; a representative must be designated by data controllers or processors not in the EU (Article 27).
DPO^c^	A person who must be designated by the controller or processor in certain circumstances (see Article 37 for more details); the duties of the DPO^c^ are defined in Article 39; they include, among others, advising the controller or processor about their duties related to the GDPR and monitoring compliance with GDPR

^a^GDPR: General Data Protection Regulation.

^b^EU: European Union.

^c^DPO: data protection officer.

### Research Background

There are several studies in the literature that have addressed the availability of privacy policies in mobile apps and the assessment of some aspects of their quality [9,15-37].

Heuristics were proposed by Hutton et al [15] to assess privacy in mHealth apps for self-tracking. In their study, Hutton et al [15] used recommendations from the US Federal Trade Commission and GDPR to define the items used in the heuristics. The evaluation of an app consists of checking its behavior and the contents of its privacy policy. They also proposed a scoring method. However, their system is not exclusively based on the GDPR and evaluates other issues that are not related to the content of the privacy policy. For example, some items in the heuristics are closely related to usability.

In 2013, Sunyaev et al [16] assessed the availability and quality of the privacy policies of the 600 most commonly used mHealth apps. They found that only 183 apps out of 600 had a privacy policy. They also determined the lengths of privacy policies, their readability, and whether privacy policies were focused on the app. Moreover, they checked if the contents of privacy policies addressed aspects users considered to be the most important. They did not develop a method or use any legal framework or regulation to design their assessment scheme.

A framework for assessing apps related to chronic insomnia disorder was defined by Leigh et al [17]. The framework was based on 24 criteria, and 6 of them dealt with privacy policies. The framework defines 6 questions about the content of the privacy policy that must be answered yes or no based on the UK Data Protection Act 1998 (and thus, Directive 95/46/EC), the UK Information Commissioner Office, and the Charter of Fundamental Rights of the EU. The authors used the answers to these 6 questions to obtain a privacy score. Blood pressure and diabetes apps were assessed by Knorr et al [18] in a study that rated 154 apps using static and dynamic analysis and evaluating web server connections and privacy policies. They assessed 12 aspects of the privacy policies including recommendations from the Organization for Economic Cooperation and Development on privacy. They discovered that 67% of the apps that stored data on the web had a privacy policy. However, they neither developed a score to evaluate privacy policies nor did they compare them.

A scoring method to evaluate the quality of 116 apps for depression was defined by O’Loughlin et al [19] using 7 questions about the privacy policy and app behavior, such as whether the app requires a personal identification number for access or the privacy policy states if data are encrypted or stored locally. Based on the results of this questionnaire, they classified privacy policies as acceptable, questionable, or unacceptable, but they did not assign a numerical score to the app, and it is not GDPR-based. They also found that only 57 out of the 116 apps they evaluated had a privacy policy.

Huckvale et al [9] assessed 79 mHealth apps, certified as clinically safe and trustworthy by the United Kingdom National Health Service Health Apps Library. They evaluated the app behavior, considering aspects like data transmission, storage, and privacy policies. When assessing privacy policies, they used a coding method based on the UK Information Commissioner Office recommendations and the UK Data Protection Act. Their proposed scheme used four domains (uses of data, technical concerns, user rights, and administrative details) to classify the topics analyzed in the privacy policy. Each of the 24 topics was classified as addressed or absent in the privacy policy. Although they discussed the percentage of apps complying with each topic, they did not develop a scoring method.

Papageorgiou et al [20] conducted a privacy and security analysis of 20 mHealth apps. They had a broad scope of analysis including static and dynamic analysis of the apps, permission analysis, and security in communications. They also investigated whether privacy policies complied with the GDPR, focusing on the right to withdraw consent, the right to portability, data protection officer (DPO) contact information, profiling, and transfers of personal data to countries not within the EU. They discussed how many apps complied with these items, but they also did not develop a scoring method.

A total of 29 apps were analyzed by Minen et al [21], focusing on data storage and privacy policies in headache apps. When analyzing privacy policies, they searched for the presence or absence of information regarding data collection, data sharing, use by children, and certain user rights.

The scientific community has been searching for a way to assess privacy in mHealth apps for the last few years. Many studies have assessed privacy in apps, usually focusing on their user interfaces, privacy in communications, and privacy policies. However, the established criteria used to analyze privacy policies are heterogeneous and subjective. These solutions are based on the researchers’ own experience, the literature, and/or an existing legal framework. The items that are considered for the assessments are very diverse, and the evaluation of these items are, on many occasions, very subjective to the evaluators’ criteria.

It is necessary, therefore, to create tools to evaluate privacy policies and establish privacy scales according to objective criteria that are less open to interpretation. Although some papers considered the GDPR, none of them proposed a set of items that enable GDPR compliance. Our aim is to fill that research gap by proposing of a GDPR-based scale to assess privacy policies in mHealth apps.

## Methods

### Privacy Scale Design

Article 13 of the GDPR summarizes the information that must be given to the user (known as data subject in the GDPR) when the information is collected from them (Table 1). This information is usually delivered to the user via a document called a privacy policy, and in order to be compliant with the GDPR, the privacy policy must meet certain requirements.

In our previous work [38], we developed a scale to assess the fairness of the privacy policies of mHealth apps. The objective of our scale was to analyze privacy policies in a systematic way and design a GDPR-based system to assess and improve such policies. Based on Article 13 of the GDPR and the recommendations of the National Data Protection Authority in Spain [39,40], we identified and summarized the information that should be provided in privacy policies (see Table 2 for a list of the items). We also defined a scoring method to assess each item.

Our scale is not intended to check strict compliance with the GDPR but to assess the fairness of privacy policies. This means that a privacy policy with fewer than 100 points (the maximum score) may be compliant, in a strict way, with the GDPR. This is because some items mentioned in Article 13 (for example, the identity of the data controller) must be analyzed carefully. It is not always easy to classify an item as yes or no in terms of compliance with the GDPR. Also, the GDPR allows some items to be omitted in certain cases, such as transfer to non-EU countries if the personal data are stored in the EU. However, our proposed score penalizes the absence of such information even if the data remain in the EU. In fact, we are just applying one of the principles of the GDPR (Article 5)—“lawfulness, fairness, and transparency”—to privacy policies, as they should go slightly further in their contents.

Indeed, the items defined in our scale may be used by developers as a checklist to design privacy policies that comply with the GDPR. They also could be used by data controllers to check if their apps are GDPR-compliant. Furthermore, a privacy policy scoring 100 points would be fully compliant with the GDPR. Using the proposed scoring method, we consider privacy policies with scores from 75.0 to 100 points as very fair (category 1 [Cat1]). A score from 50.0 to 74.9 is somewhat fair (category 2 [Cat2]). A score from 25.0 to 49.9 is somewhat unfair (category 3 [Cat3]). Finally, we consider a score from 0 to 24.9 as very unfair (category 4 [Cat4]).

Some discrepancies in the interpretation of how to assign a score to some items were found in the first iteration of the scale design process. In the first iteration, the privacy policies of 9 apps were analyzed, and we obtained Kappa-Cohen indexes for each item. Possible scores for each item and Kappa-Cohen indexes are shown in Table 3.

**Table 2:** Items in the privacy policy (Article 13).

Item	Item number
Identity of data controller	1
Identity of the representative	2
Data protection officer details	3
Purposes for the processing	4
Legal basis for the processing	5
Legitimate interests from controller	6
Recipients (or categories) of the personal data	7
Transfers to non–European Union countries	8
Period for which data will be stored	9
Existence of data subject’s rights	10
Existence of right to withdraw consent	11
Right to lodge a complaint with a supervisory authority	12
Obligation to provide personal data	13
Existence of automated decision making or profiling	14

**Table 3:** Kappa-Cohen indexes for privacy policy items for the 9 apps evaluated in the first iteration.

Item	Item number	Score	Kappa-Cohen index (n=9)
Identity of data controller	1	0: no info; 0.5: partial; 1: full	0.77
Identity of the representative	2	0: no info; 1: info provided; N/A: not applicable	1
Data protection officer details	3	0: no info; 1: info provided	0.61
Purposes for the processing	4	0: no info; 0.5: generic; 1: specific	0.77
Legal basis for the processing	5	0: no info; 1: info provided	0.77
Legitimate interests from controller	6	0: no info; 1: info provided; N/A: not applicable	0.8
Recipients (or categories) of the personal data	7	0: no info; 1: info provided	–0.13
International transfers of data	8	0: no info; 0.5: generic; 1: full details or no international transfers	0.53
Period for which data will be stored	9	0: no info; 0.5: generic; 1: specific	0.66
Existence of data subject’s rights	10	0: no info; 0.5: generic; 1: full	0.49
Existence of right to withdraw consent	11	0: no info; 1: info provided; N/A: not applicable	0.08
Right to lodge a complaint with a supervisory authority	12	0: no info; 0.5: generic; 1: specific	0.77
Obligation to provide personal data	13	0: no info; 1: info provided	–0.17
Existence of automated decision making or profiling	14	0: no info; 0.5: generic; 1: specific or no profiling or automated decision making done	0.17

A refinement of the criteria used to assign those scores was performed to resolve the discrepancies. Also, an error in the definition and description of one of the items was corrected. Items and their possible scores are (re)defined as follows:

- Identity of data controller: 1 point if full information is given. Full information means name, postal address, and electronic address (both email and a contact form are considered valid) of the data controller; 0.5 points if some information is missing; 0 points if the information is omitted. If only an electronic address is provided, the score is 0 points. Also, if the street address is not mentioned, the score is 0 points.
- Identity of the representative: The representative is a natural or legal person, established in the EU, who must be designated by the data controller if they are not in the EU. In this case, 1 point is given if full information is given (in the same way as with the data controller) and 0 points otherwise.
- DPO details: The GDPR states that a DPO must be designated if the controller processes a large quantity of data in some special categories, such as health data. We assume that a DPO must exist in any given mHealth app. At least an email address must be given to get 1 point. In order to be consistent with the definition of DPO in the GDPR, the DPO must be a different person from the data controller, so the email address should also be different. Otherwise, 0 points are given.
- Purposes for the processing: The purposes for the processing must be stated explicitly in the privacy policy. Sometimes the information given is too general. For example, “We collect this information for the purpose of providing our service” does not give any detail about why the data controller needs the personal data. In this case, the score for this item is 0.5 points. If purposes are provided explicitly, 1 point is given. If purposes are not mentioned, 0 points.
- Legal basis for the processing: There are six legal bases for the processing (Article 6 of GDPR): consent, need to perform a contract, legal obligation, protect vital interest of somebody, public interest or exercise official authority, and legitimate interest. This information must be given in the privacy policy in order to get 1 point. However, we found that, in some cases, the legal basis in which the processing was founded was not explicitly stated in a separate paragraph. This is because, sometimes, this information is embedded in the declaration of the purposes for the processing. As the information is, after all, given to the data subject, we decided to give 1 point in these cases. Furthermore, if 0 points are given to this indicator, indicators based on the legal basis (ie, items 6 and 11) are considered N/A (not applicable).
- Legitimate interests from controller: 1 point if this information is given and 0 points otherwise. N/A if legitimate interest is not stated as a legal basis for the processing or if item 5 is 0 points.
- Recipients (or categories of recipients) of the personal data: 1 point if this information is given and 0 points otherwise. We must note that if there are no recipients, this must be explicitly stated. Also, we must keep in mind that in accordance with Article 4 of the GDPR a data processor is considered a recipient.
- Transfers to non-EU countries: This item refers to the fact that personal data may be transferred to a country not in the EU. In this case, the data controller must give enough information about the measures that are in place to achieve a similar level of protection. We give 1 point if privacy policy indicates that this transfer is in place, and there is a reference to the measures taken. We consider enough information to contain a mention to the compliance with Privacy Shield [14] or similar frameworks. We also give 1 point if the transfer is based on an adequacy decision from the Commission. If there is a mention to a transfer to non-EU countries without further information, this item is 0.5 points. The GDPR states that this information must be given if there are transfers to non-EU countries and says nothing if the data are stored within the EU. We consider that this information must be given even where there are no transfers to non-EU countries. Thus, if there is no information about transfers outside the EU, this item is 0 points. When the data controller is in the EU, the fact of not transferring data outside the EU must be explicitly stated. If not, the item is 0 points. Otherwise, the item is N/A.
- Period for which data will be stored: To obtain 1 point, the privacy policy must point out a specific time in the future when the data will be erased. We consider the following time references as valid: a period of inactivity in the data subject’s account or a specific reference to a user request to erase the data. The latter is independent of the specification of data subject’s rights in the privacy policy.
- Existence of data subject’s rights: 1 point is given if the specific user’s rights mentioned in Article 13 (right to access, rectification, erasure, restriction of processing, object of processing, and data portability) are enumerated in the privacy policy with a way to exercise these rights. This information may also be provided using a link. We award 1 point if 5 or more rights are mentioned and 0.5 points if partial information is given (for example, some rights are omitted or there is no indication on how to exercise the rights); 0 points if there is no reference to data subject’s rights.
- Existence of the right to withdraw consent: This is an additional right that only exists if the legal basis for the processing is consent. As such, the score is 1 point if this right is mentioned (along with the way to exercise it) and 0 points if the right is omitted. This item is N/A if consent is not one of the legal bases for the processing or if item 5 is 0 points.
- Right to lodge a complaint with a supervisory authority: According to the GDPR, there is an obligation to inform users that they have the right to lodge a complaint with a supervisory authority if they believe that their rights have been violated. To score 1 point, the privacy policy must not only identify the appropriate supervisory authority but also provide at least a link to it. Moreover, 1 point is given if the policy links to the list of all supervisory authorities within the EU. Simply naming the supervisory authority is considered insufficient and earns a score of 0.5 points and 0 points otherwise.
- Obligation to provide personal data: The privacy policy must explicitly state what happens if the user does not provide certain personal data. Examples of good practices are the following: “if you choose not to provide data, we may not be able to provide you those services” and “In order to join [...] you must provide [...].” If this information is given, 1 point. Otherwise, 0 points.
- Existence of automated decision making or profiling: We consider that there must be a reference to the existence or absence of automated decision making or profiling based on personal data. This item may be tricky to interpret because sometimes apps make automated decisions such as defining user interface language based on personal data. This example probably does not fit within the reasoning of the GDPR. Thus, we consider as automated decision making or profiling a behavior that goes beyond simple decisions made by the app. This item scores 1 point if this information is shown in the privacy policy and enough information about the logic around this decision or profiling is given. We consider a link to the information to be valid. Therefore, 0.5 points are scored if there is a reference to this item with no additional information, and the score is 0 points if no information is given. Simply using Google Analytics is not sufficient to consider that the app to be profiling. However, the use of cookies, if they modify the app behavior, is considered profiling. We are also aware that, in accordance with the National Data Protection Authority in Spain on mobile apps [41], some information must be given if the app includes targeted advertisements.

As stated in the introduction, an important objective of our research is to provide a reproducible scale for the assessment of privacy policies in mHealth apps, so schematics and further descriptions of the items with explanatory examples for the scoring system are defined in Multimedia Appendix 1.

The proposed scale consists of 14 items to check when assessing a privacy policy, as shown in Table 1. Each item is assigned a score of 0 points or 1 point, though some of them may score 0.5 points. Thus, every item has the same weight within the total score. Also, as some items may be N/A, the final score is expressed as a percentual score. Thus, if an app achieves 7 points when privacy items are assessed but only 12 items are applicable, its final score is 58.3 points. Like the scales previously defined in the literature, our scale must be simple and easy to apply. Our scoring method is in concordance with other mHealth app privacy scales [15,27,32], which are also composed of yes/no questions. Most of the privacy scales that defined a score were based on items that could have 2 or 3 values.

Items 1, 4, 8, 9, 10, 12, and 14 can score 0.5 points, as some items are more complex than others. For example, the item purposes for the processing requires that the data controller be very explicit when defining the app’s purposes for the processing. We found that some data controllers state the purposes for the processing but are not as explicit as they should be. In these cases, the item scores 0.5 points. Other items, such as item 3 (DPO details) are so simple that they can only score 1 point (yes) or 0 points (no). Additional details and examples can be found in Multimedia Appendix 1.

We have also added new indicators that did not appear in our previous work. They do not directly influence scores, but they add some information that is relevant to the study of privacy policies:

- Date of last update of the privacy policy: We look for this information in the text of the privacy policy itself. We do not consider alternative ways of obtaining the last update date such as analyzing http last-modified headers [42].
- Data controller’s country: We collect this from the identity of the data controller. We do not consider additional information such as any obtained by the WHOIS tool [43]. This tool provides information about the domain name, but it might be misleading.
- GDPR awareness: This indicator only gets a yes/no value indicating whether GDPR is explicitly mentioned in the privacy policy.

### Case Study: Cancer Apps

#### Study Design

A systematic search strategy was followed to identify all relevant mHealth apps for the most common types of cancer (breast, prostate, colorectal, and lung cancer) and for cancer in general. We focused on Android apps due to its market dominance, being the most installed operating system among the new smartphones shipped worldwide from 2017 to 2019 [44]. Two researchers (ORR and EDZ) searched the Spanish version of the Google Play website, taking steps to ensure that no previous searches or cookies influenced the results. Five searches were completed on July 25, 2019, using “cancer mama,” “cancer prostata,” “cancer,” “cancer colon recto,” and “cancer pulmon” as search strings.

#### Selection Criteria

Apps were included in the screening stage if their title or description contained one of the search strings defined. After duplicates were removed, two researchers (ORR and EDZ) reviewed and assessed the title and description of the resulting mHealth apps for eligibility against the selection criteria. Apps whose titles and/or descriptions met the selection criteria were downloaded and installed. A researcher (ORR) checked that they worked properly and met the selection criteria. Disagreements were resolved by consensus.

The following inclusion criteria were used: the title or description referred to at least one of the search strings, it was intended exclusively for cancer patients or survivors, and the app collected user data or allowed users to share their opinions or data.

Apps were excluded if they met at least one of the following conditions: the title or description was written neither in English nor Spanish, the user interface was available neither in English nor Spanish, the privacy policy was written neither in English nor Spanish, it was not focused on cancer, it was intended for people other than cancer patients or survivors, or it was not free.

#### Data Extraction

The following descriptive characteristics of apps meeting the selection criteria were collected from the Google Play website when available: developer, category, number of ratings, user rating, last update, and number of downloads. Additionally, using the information included in the description, two researchers (ORR and EDZ) independently classified the apps according to main purpose and type of cancer. Discrepancies were resolved by consensus.

URLs linking to the privacy policy of the included apps were also collected when available. If no link was provided, we tried to find the privacy policy on the developer’s website. A researcher (JR) reviewed all installed apps and checked if they contained any additional privacy policy information. If the privacy policy contained in the app was different from the one linked in the Google Play website, the former was considered for the assessment. When user registration was required, the privacy policy had to be available before registering for the service. Otherwise, we considered the app to not have a privacy policy, as it was not accessible before using the app. Two researchers (JB and JR) reviewed and independently assessed all privacy policies of the included apps using the proposed scale. Finally, a score for each privacy policy was assigned according to the scoring scale defined earlier. Discrepancies were resolved by consensus.

#### Classification

Included apps were classified according to main purpose and type of cancer. We used the classification scheme for an app’s main purpose proposed in Giunti et al [7]. We removed the awareness-raising option because the apps designed for this purpose did not meet the selection criteria and were excluded from our study. Therefore, we coded an app’s main purpose as disease and treatment information (DTI), disease management (DM), or support (S).

Finally, type of cancer was coded as general, colorectal, breast, prostate, lung, or other. General was used to code included apps that pertained to cancer in general without identifying any specific type. Other was assigned to apps that pertained to a specific type of cancer other than colorectal, breast, prostate, or lung cancer.

## Results

### App Selection and Extracted Features

**Figure 1:**
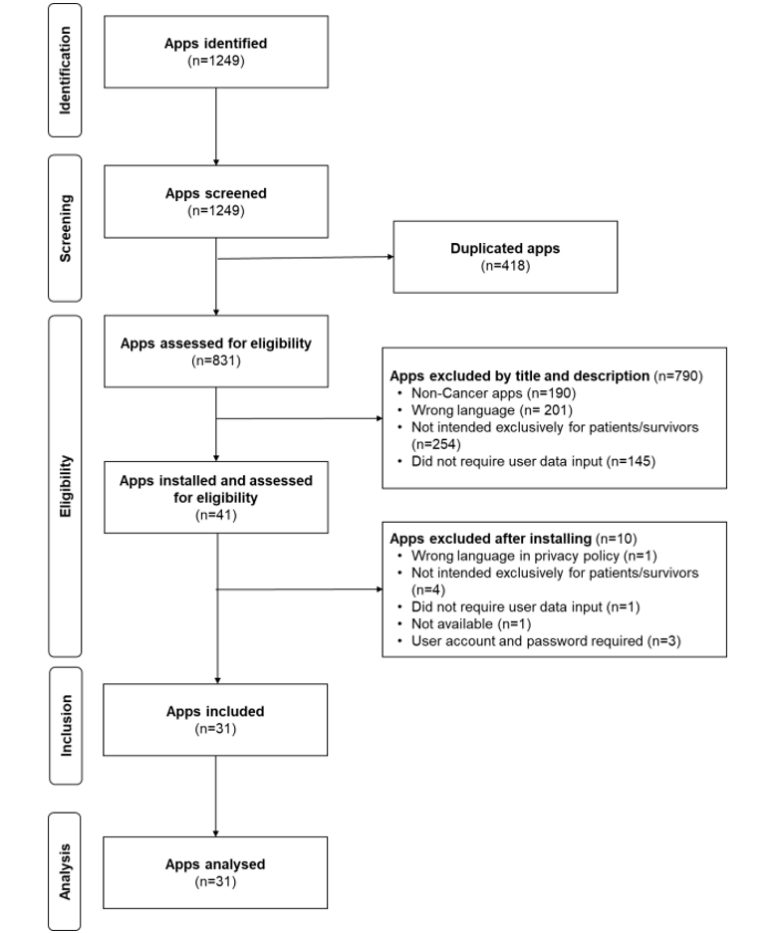
Flow diagram.

Google Play searches resulted in 1249 mHealth apps. After duplicates were removed, 831 mHealth apps were assessed for eligibility; 41 of those apps met the selection criteria and were downloaded and installed on an Android smartphone (Moto G7, Motorola Mobility, LLC) to check if they worked properly. Finally, 31 mHealth apps met the selection criteria and were included in the analysis. Figure 1 shows the flow diagram of the described procedure, while Table 4 shows the selected apps considering the selection criteria. For convenience when analyzing the results, apps were tagged from App1 to App31. For the statistical analysis below, five more features were added to Table 4: Google Play app ratings, number of reviews, number of downloads, app type, and cancer type. Multimedia Appendix 2 contains a list of the apps that were found in Google Play search and those included in the case study.

We applied the second iteration of our privacy policy assessment scale (described in the Methods section) to the 31 selected apps. In the case of App15, a privacy policy link led to a generic privacy policy for the company, so we considered the one in the app. Table 5 shows the privacy scores obtained after assessing all the privacy policies. We also added more information about the apps to the table in order to achieve a more complete analysis of the results. In particular, we added the data controller’s location, the last app update, and whether the privacy policy was GDPR-aware (ie, it mentioned the GDPR). If the app did not have a privacy policy, it was assigned a score of 0 points.

**Table 4:** Selected apps.

App name	Developer	Rating (stars)	# Ratings	# Downloads	App type	Cancer type	Label
BECCA: Breast Cancer Support	Breast Cancer Care	4.5	63	10,000+	S^a^	Breast	App1
EmotionSpace cáncer de mama	Pfizer Inc	2.5	2	100+	S	Breast	App2
ChemoWave: For Cancer Patients	Treatment Technologies & Insights	4.4	20	1000+	DM^b^	General	App3
OWise Breast Cancer	Px HealthCare BV	4.4	10	1000+	DM	Breast	App4
My Cancer Coach	Genomic Health Inc	4.5	86	10,000+	DM	General	App5
Breast Advocate	Toliman Health	5	1	100+	DTI^c^	Breast	App6
Breast Cancer Support	MyHealthTeams	4.1	47	1000+	S	Breast	App7
KMBCN	Kepharge	5	1	10+	DTI	Breast	App8
Triple Negative Breast Cancer	Kognito	5	2	100+	DTI	Breast	App9
Breast Cancer: Others Like Me	Eli Malki	0	0	5+	S	Breast	App10
Outcomes4Me	Outcomes4Me Inc	5	5	100+	DTI	Breast	App11
Boobytrapp: The Breast Cancer App	Boobytrapp	3.7	3	100+	S	Breast	App12
The BAPS App Wales	The Orchard Media & Events Group Ltd	0	0	100+	DM	Breast	App13
BELONG Beating Cancer Together	BelongTail	4.7	1,151	100,000+	DM	General	App14
Diana	F Hoffmann–La Roche	5	7	1000+	DM	Breast	App15
Got Boobs?	Got Boobs	0	0	100+	S	Breast	App16
inKind Space	PixelEdge	0	0	10+	S	Breast	App17
Cancer Surveillance	GoMLV	3.7	21	1000+	DM	General	App18
Focalyx	Lyx Health	4.8	6	50+	DM	Prostate	App19
Adrenal Cancer: Others Like Me	Eli Malki	5	6	1000+	S	Other	App20
How Are You Today? PC	Intelesant	0	0	100+	DM	Prostate	App21
Cancer.Net Mobile	American Society of Clinical Oncology	4.2	227	10,000+	DM	General	App22
TNM Cancer Staging	International Atomic Energy Agency	4.6	323	10,000+	DTI	General	App23
Untire: Beating cancer fatigue	Tired of Cancer BV	4.5	60	5000+	DM	General	App24
Self-Care During Cancer	NearSpace Inc	4.7	6	1000+	S	General	App25
CanDi: Cancer Diet App	Faculty of Health Sciences UniSZA	4.7	60	500+	DM	General	App26
CancerAid	CancerAid PTY LTD	3.7	25	1000+	DM	General	App27
GRYT Health Cancer Community	GRYT Health	3.9	7	100+	S	General	App28
Target Ovarian Cancer Symptoms Diary	Brandwave Marketing	3.6	8	1000+	DM	Other	App29
Pancreatic Cancer Action: Symptom Tracker	Healthbit Ltd	5	3	100+	DM	Other	App30
My Care Plan (cancer survivor)	NearSpace Inc	4	4	1000+	DM	General	App31

^a^S: support.

^b^DM: disease management.

^c^DTI: disease and treatment information.

**Table 5:** Privacy scores.

App name	Label	Data controller’s location	Last update	GDPR^a^ aware	Score
BECCA: Breast Cancer Support	App1	UK^b^	03/2019	No	76.9
EmotionSpace cáncer de mama	App2	Germany	05/2018	No	75
ChemoWave: For Cancer Patients	App3	US^c^	10/2018	No	53.6
OWise Breast Cancer	App4	UK	N/A^d^	Yes	31.8
My Cancer Coach	App5	US	02/2015	No	23.1
Breast Advocate	App6	Unknown	No privacy policy	N/A	0
Breast Cancer Support	App7	US	09/2019	Yes	78.6
KMBCN	App8	Unknown	No privacy policy	N/A	0
Triple Negative Breast Cancer	App9	US	02/2019	No	34.6
Breast Cancer: Others Like Me	App10	Unknown	No privacy policy	N/A	0
Outcomes4Me	App11	Unknown	11/2018	No	34.6
Boobytrapp: The Breast Cancer App	App12	Singapore	06/2018	No	29.2
The BAPS App Wales	App13	UK	N/A	Yes	69.2
BELONG Beating Cancer Together	App14	Israel	09/2018	Yes	75
Diana	App15	Spain	10/2018	No	40.9
Got Boobs?	App16	US	10/2018	No	26.9
inKind Space	App17	US	N/A	No	25
Cancer Surveillance	App18	Unknown	N/A	No	15
Focalyx	App19	Unknown	No privacy policy	N/A	0
Adrenal Cancer: Others Like Me	App20	Unknown	No privacy policy	N/A	0
How Are You Today? PC	App21	Unknown	No privacy policy	N/A	0
Cancer.Net Mobile	App22	US	07/2019	Yes	50
TNM Cancer Staging	App23	Unknown	No privacy policy	N/A	0
Untire: Beating Cancer Fatigue	App24	Netherlands	N/A	Yes	66.7
Self-Care During Cancer	App25	US	03/2014	No	29.2
CanDi: Cancer Diet App	App26	Unknown	No privacy policy	N/A	0
CancerAid	App27	Australia	N/A	No	42.9
GRYT Health Cancer Community	App28	US	12/2018	No	46.2
Target Ovarian Cancer Symptoms Diary	App29	UK	04/2018	Yes	80.8
Pancreatic Cancer Action: Symptom Tracker	App30	UK	06/2018	Yes	75
My Care Plan (cancer survivor)	App31	Unknown	No privacy policy	N/A	0

^a^GDPR: General Data Protection Regulation.

^b^UK: United Kingdom.

^c^US: United States.

^d^N/A: not applicable.

### Assessment of Privacy Policies

**Figure 2:**
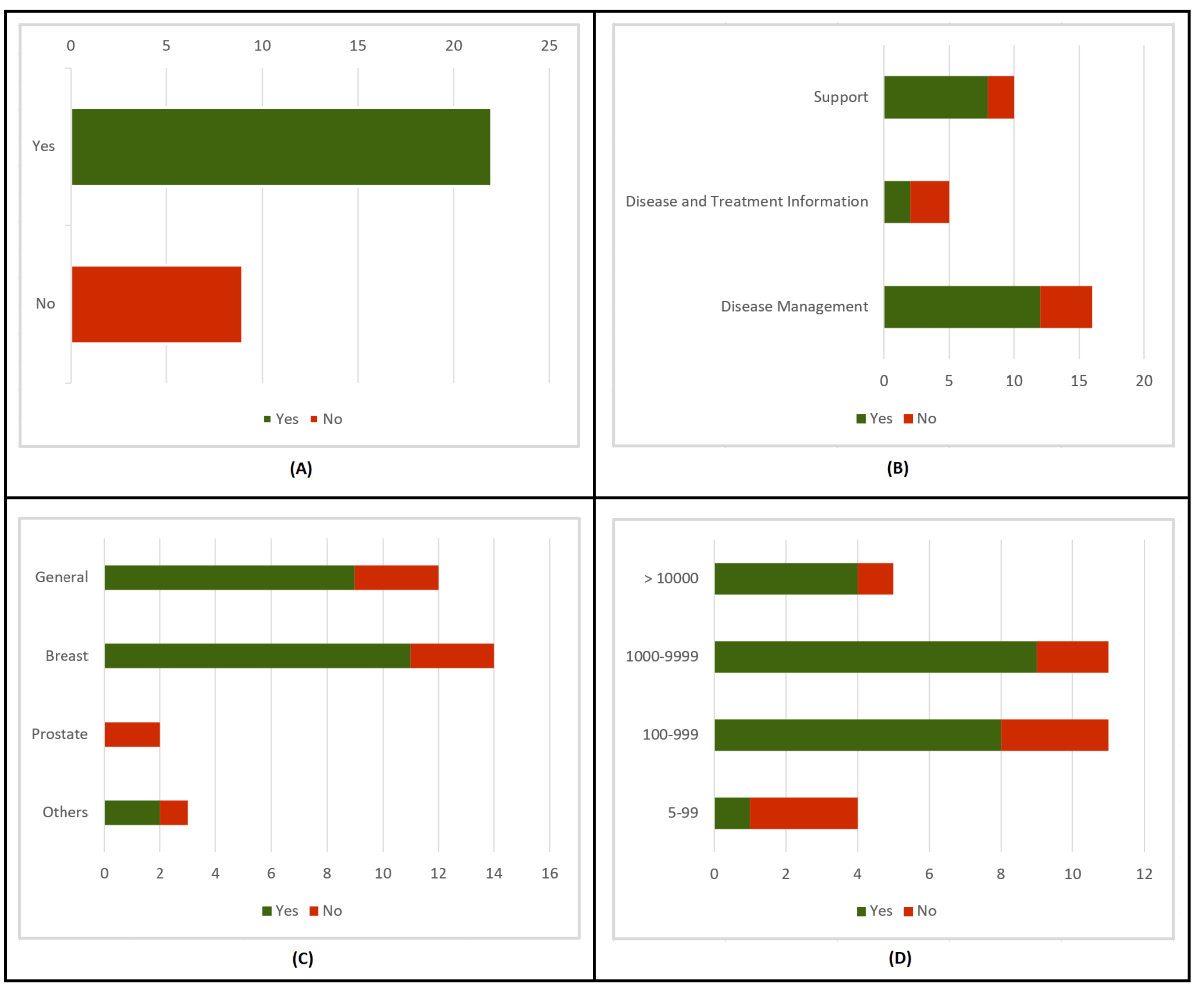
Analysis of privacy policy presence.

**Figure 3:**
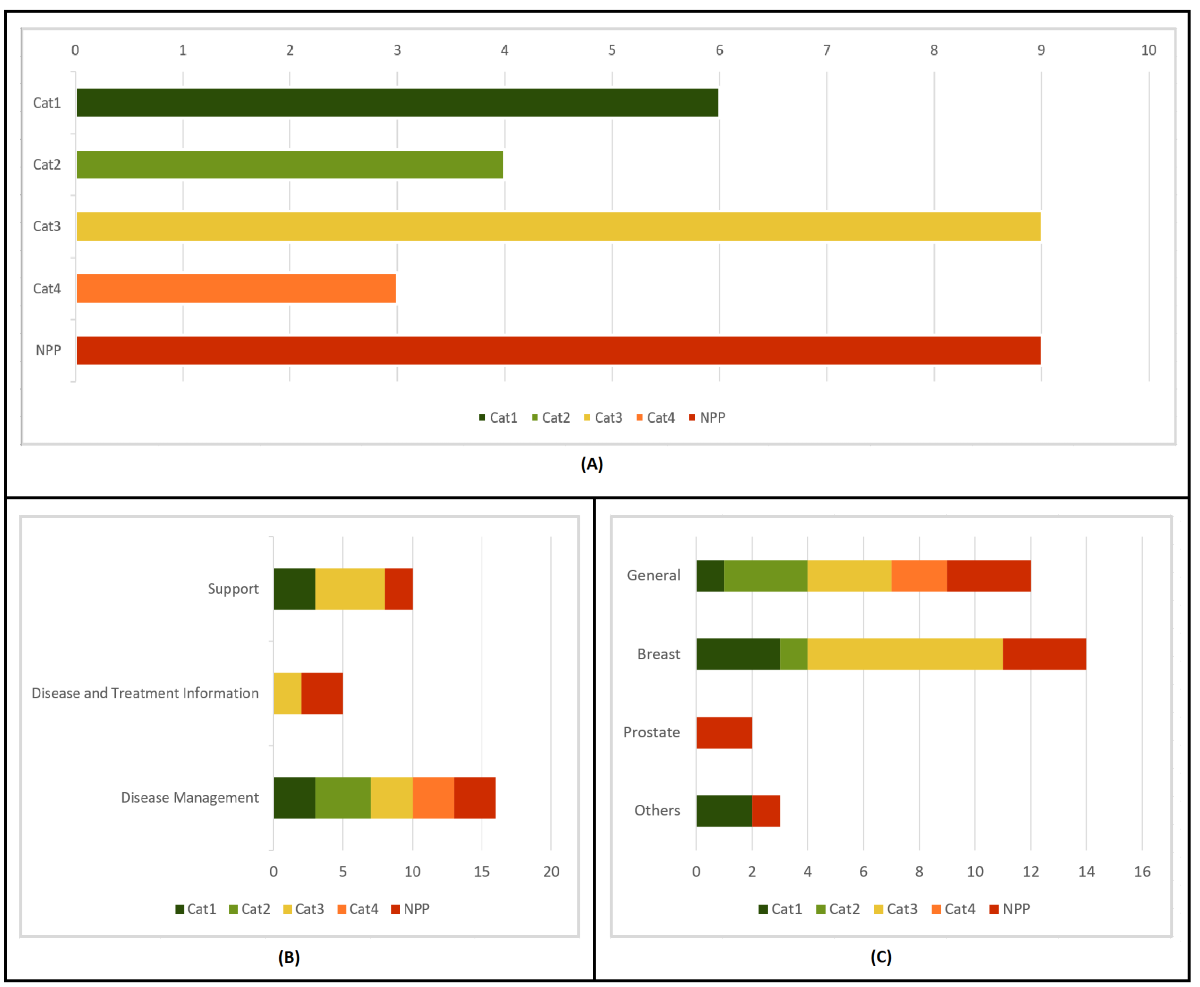
Privacy score summary (part1).

**Figure 4:**
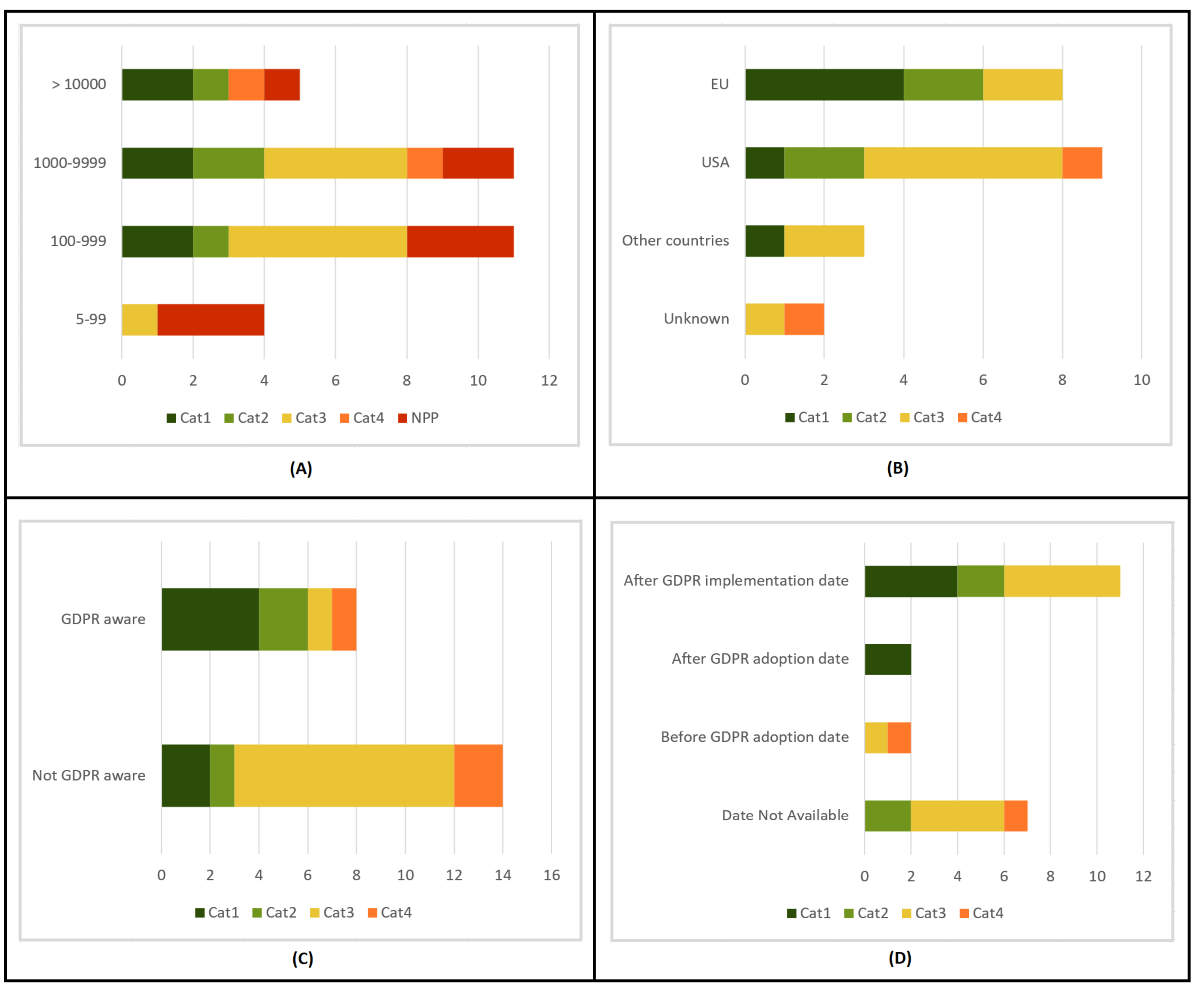
Privacy score summary (part2).

We assessed the fairness of the privacy policies of 31 cancer apps. Surprisingly, as seen in Figure 2A, 29% (9/31) of the included apps did not have a privacy policy. Thus, we first determined the presence/absence of a privacy policy according to different features in the apps. We considered the app type, the type of cancer, and the number of downloads. A summary of the analysis is shown in Figure 2. Second, we analyzed privacy policies according to the obtained score. As specified in the Methods section, Cat1 (75.0 to 100 points), Cat2 (50.0 to 74.9 points), Cat3 (25.0 to 49.9 points), and Cat4 (0 to 24.9 points). Only 19% (6/31) of apps had a Cat1 privacy policy, while 4 apps belonged to Cat2. Thus, only 32% (10/31) of apps scored above 50.0 points in our GDPR-based privacy policy assessment. The results are shown in Figure 3A, where NPP means no privacy policy. We also analyzed the fairness of privacy policies according to different features in the apps, considering the app type, type of cancer, and number of downloads. When assessing fairness, we also considered the data controller’s country, last privacy policy update, and if the privacy policy was GDPR-aware. A graphic summary of privacy scores can be seen in Figure 3 and Figure 4.

Privacy policy fairness of 4 included apps had been previously analyzed in Benjumea et al [38]: App1, App4, App7, and App12. Despite the minor modifications in the scale for the second iteration, App4, App7, and App12 did not change their scores (even though App7 had recently updated its privacy policy). App7’s score was one of the highest, with 78.6 points. More remarkable is the case of App1: with a recent update, its score went from 57.7 to 76.9 points. This indicates a significant effort to follow GDPR guidelines.

#### Assessment of Privacy Policies by App Type

We analyzed the presence of privacy policies according to app type and found that 75% (12/16) of disease management apps and 80% (8/10) of support apps had a privacy policy. As seen in Figure 2B, only 40% (2/5) of disease treatment and information apps had a privacy policy. Regarding the score, 44% (7/16) of disease management apps were above 50 points. Only 30% (3/10) of support apps were above 50 points, but all of them were in Cat1. No data treatment and information app reached a score of 50. These results are presented in Figure 3B.

#### Assessment of Privacy Policies by Type of Cancer

Regarding the presence of a privacy policy according to cancer type, both general cancer apps (9/12, 75%) and breast cancer apps (11/14, 79%) had better results than prostate cancer (0/2). However, we consider that only 2 prostate cancer apps are not representative enough for a further analysis. As for other cancer apps, 67% (2/3) had privacy policies. Results are displayed in Figure 2C. In all, 3 of the breast cancer apps and 1 general cancer app were in Cat1, while the two other type apps that had a privacy policy were also above 75 points. These results do not seem to permit the drawing of any definite conclusions about the relationship between privacy policies and the type of cancer. Results can be seen in Figure 3C.

#### Assessment of Privacy Policies by Number of Downloads

Next, we evaluated the relationship between number of downloads and the presence of a privacy policy. We gathered apps into 4 groups: 5 to 99 downloads, 100 to 999 downloads, 1000 to 9999 downloads, and more than 10,000 downloads. The last 3 groups yielded similar results: 80% (4/5), 82% (9/11), and 73% (8/11), respectively, had a privacy policy. The only significant difference was observed in apps with 5 to 99 downloads: only 25% (1/4) had a privacy policy. These results can be seen in Figure 2D. There was, however, a difference in scores. None of the apps with 5 to 99 downloads reached 50 points. As for apps with 100 to 999 and 1000 to 9999 downloads, only 27% (3/11) and 36% (4/11) of apps, respectively, were in the first two categories. A total of 60% (3/5) of apps with more than 10,000 downloads were above 50 points. Thus, we found a clear relationship between the number of downloads and the average fairness of privacy policies, which can be observed in Figure 4A.

#### Assessment of Privacy Policies by Data Controller’s Country

Three more features were analyzed but only in cases where a privacy policy was present. The first was the data controller’s country. We identified 4 groups: EU apps (8), US apps (9), apps from other countries (3), and apps from unknown countries (2). According to our analysis, the fairness of EU privacy policies was much better than those from the United States, with 75% (6/8) of EU apps above 50 points, and 4 of them in the first category. Meanwhile, only 33% (3/9) of US apps were in the first two categories. Only 33% (1/3) from other countries were above 50 points. As for the two apps for which the data controller’s country was unknown, neither reached 50 points. These results can be seen in Figure 4B.

#### Assessment of Privacy Policies by General Data Protection Regulation Awareness

The second feature that was analyzed when a privacy policy was present was GDPR awareness. When the GDPR was explicitly mentioned in the app’s privacy policy, 75% (6/8) of those apps reached the first two categories. Moreover, half of them reached the first category. When the GDPR was not mentioned, only 21% (3/14) reached 50 points. Figure 4C demonstrates this.

#### Assessment of Privacy Policies by Last Update

Finally, last privacy policy update was evaluated. We identified 4 groups: apps updated after GDPR implementation (May 25, 2018), apps updated after GDPR adoption (April 14, 2016), apps updated before GDPR adoption, and unknown update date. In fact, half of the 22 apps with a privacy policy were updated after GDPR implementation. Two more were updated after GDPR adoption. We found that 62% (8/13) of the apps updated after GDPR adoption were in the first two categories. Meanwhile, only 22% (2/9) of the apps not updated after GDPR adoption reached 50 points. Thus, update recency seems to be related to app privacy policy fairness. Figure 4D shows the results.

#### Assessment of Privacy Policies by Popularity

Last, we analyzed how popularity affects fairness in privacy policies. Popularity in an app is often measured by the number of downloads and the number of stars [45]. Consistent with previous literature, apps with fewer than 10 ratings were excluded to avoid unfair ratings [46]. Table 6 shows the scores for the apps that have been rated more than 10 times, ordered by their number of stars. Evidently, it is difficult to find any relationship between popularity and privacy score. The apps ranked second and third did not even have a privacy policy, even though App23 had more than 300 ratings. Moreover, App5 was ranked fourth and had a Cat4 privacy score.

**Table 6:** Assessment of privacy policies by app popularity.

App label	Stars	Ratings	Downloads	Privacy score
App14	4.7	1151	100,000+	75
App26	4.7	60	500+	0
App23	4.6	323	10,000+	0
App5	4.5	86	10,000+	23.1
App1	4.5	63	10,000+	76.9
App24	4.5	60	5000+	66.7
App3	4.4	20	1000+	53.6
App4	4.4	10	1000+	31.8
App22	4.2	227	10,000+	50
App7	4.1	47	1000+	78.6
App27	3.7	25	1000+	42.9
App18	3.7	21	1000+	15

### Analysis of Item Compliance

Finally, we analyzed item compliance for the 22 apps with a privacy policy. Table 7 summarizes the results. We see a heterogeneous compliance of the different items that we checked. Only a few of the items mostly complied. Item 7, showing the recipients of personal data, was satisfied by 95% (21/22) of apps, while item 4, regarding the purposes of processing, was satisfied by 91% (20/22) of apps, with the other two giving partial information. Another item with positive results was item 1: 77% (17/22) of apps provided the identity of the data controller, with 3 more apps giving partial information. The last positive item was item 5. A total of 68% (15/22) of apps determined a legal basis for the processing.

Three items showed varied behavior. For item 10, 45% (10/22) of apps showed the existence of data subject’s rights, with 2 more apps giving partial information. For both items 8 and 9, 36% (8/22) of apps disclosed transfers to other countries and about the period of personal data storage. Some apps gave partial information about them.

A negative behavior was observed for the rest of the items. Only 27% (6/22) of apps satisfied items 3 and 13. Item 3 regarded the DPO’s contact details, while item 13 dealt with the obligation of providing personal data and the possible consequences of not providing such data. A total of 6 apps did not comply with item 11, which regarded the right of withdrawing consent at any time. However, item 11 was not applicable in 8 apps, as consent was not a legal basis for data processing. We determined that 23% (5/22) of apps satisfied item 12, the right to lodge a complaint with a supervisory authority, with 3 more apps giving partial information. Item 6 is quite particular, as it is only applicable when the legitimate interests of the data controller constitute the legal basis for the processing. Only 33% (3/9) of apps complied with it, while it was not applicable in 13 apps. Item 14 was satisfied by only 9% (2/22) of apps. Information about profiling was not available in most of the cases. Last, none of the 13 apps outside the EU complied with item 2. The apps outside the EU should provide the identity of a representative inside the EU. This item was not applicable to the 9 apps in the EU.

**Table 7:** Summary of compliance with General Data Protection Regulation items.

Item number	Full information	Partial information	No information	Not applicable
1	17	3	2	0
2	0	0	13	9
3	6	0	16	0
4	20	2	0	0
5	15	0	7	0
6	3	0	6	13
7	21	0	1	0
8	8	7	6	13
9	8	5	9	0
10	10	2	10	0
11	6	0	8	8
12	5	3	14	0
13	6	0	16	0
14	2	2	18	0

## Discussion

### Principal Findings

This paper proposes a GDPR-based scale for assessing the fairness of privacy policies. We defined 14 items that provide developers with a tool to comply with the GDPR, while data controllers and users can use the scale to obtain a score that defines the fairness of privacy policies. Countries are really starting to be concerned about privacy and its implications. This has led the EU to develop laws that help protect user privacy. As a result, the GDPR was adopted in April 2016 and implemented in May 2018. In this study, we developed the second iteration of our GDPR-based method to assess the fairness of privacy policies. In this iteration, we refined the scores and criteria to obtain such scores with the aim of assessing not only compliance with the GDPR but fairness of the privacy policies in an objective way. Discrepancies between researchers have been critically reduced. A percentual score was defined, with a maximum of 100 points. A low score indicated not necessarily that an app did not comply with the GDPR but could indicate a low fairness of its privacy policy.

As privacy is crucial in mHealth and particularly for cancer patients, we assessed 31 Android cancer apps from Google Play. In order to foster straightforward interpretation of the results, we classified scores into four categories according to their fairness (from most to least fair): Cat1, Cat2, Cat3, and Cat4. We analyzed the fairness of privacy policies by app type, cancer type, number of downloads, data controller country, GDPR awareness, and last privacy policy update.

The first disappointing result was the absence of a privacy policy in 9 of 31 apps. This means that only 71% of the apps had a privacy policy. According to the literature, when top mHealth apps were analyzed, the percentage of apps with a privacy policy was about 90% [22,23]. However, when the type of app was selected, results were similar to ours: according to Bondaronek et al [32], 75% of physical activity apps had a privacy policy, while in Adhikari et al [33], they found that 69% of depression and smoking cessation apps had a privacy policy. When the selection is smaller, results are even worse. O’Loughlin et al [19] found privacy policies in only 49% of depression apps, while Sunyaev et al [16] showed that, surprisingly, 31% of medical or health and fitness apps had privacy policies. Moreover, when we analyzed the scores according to the type of app, it was noticeable that disease management apps and support apps obtained better scores than disease treatment and information apps. We believe that this is a positive fact, as disease management apps and support apps handle more sensitive information about patients.

In the literature, there are two ways to assess privacy. Some articles evaluated the different apps according to several items, eventually obtaining a score [27,28,36], while others checked if the analyzed apps met the criteria they had defined [20,32,37].

Regarding scores, only 45% of apps with a privacy policy that we assessed had a score greater than or equal to 50%, with an average score of 50.5 points. Only Hutton et al [15] had a comparable scoring system. They built a 26-item heuristic to assess privacy in mHealth apps, although only the first 7 items dealt with privacy policies. They applied their heuristic to 64 self-tracking mHealth apps and found an average score of 46.2%, with a high dispersion. Thus, their results are quite in line with ours.

The most complied-with items were the following: item 1 (identity of data controller), item 4 (purposes for the processing) and item 7 (recipients or categories of the personal data). Still, only 45% (5/22) of apps fully informed users about their rights (item 10) and only 5 fully informed users about their right to lodge a complaint with a supervisory authority. Finally, it is interesting that none of the 13 apps whose data controller was not within the EU informed users of the identity of their representative in the EU.

It is difficult to find such a complete analysis in the literature, but some of the items were assessed by different articles. Item 1 was evaluated in Hutton et al [15] and Papageorgiou et al [20], which were complied with by 75% and 63% of apps, respectively. Results were similar to our study, where 77% of apps satisfied this item. Item 1 was also analyzed in Huckvale et al [22], but results were very different. Only 25% of apps identified the data controller. Item 3 was also evaluated in Papageorgiou et al [20], with none of the apps having a DPO. Our study showed that 27% of apps had a DPO. Item 4 was assessed in Hutton et al [15] and Minen et al [21]: 61% and 64% of apps complied with this item, respectively, whereas a better result (91%) was obtained in this paper. Item 7 was assessed in Hutton et al [15], with 61% of apps stating the recipients of personal data; 96% of apps makes item 7 the most complied-with item in our study. Item 9 was evaluated in Huckvale et al [22]: 32% of apps stated the period for which personal data will be stored, compared with 36%. In Minen et al [21], item 10 was analyzed: 36% of apps informed users about their rights, whereas we obtained a result of 46%. Item 11 was assessed in Hutton et al [15] and Papageorgiou et al [20]: 55% and 37% of apps complied with this item, respectively. In our study, 43% of apps informed users about the right to withdraw consent. Items 12 and 13 were assessed in Huckvale et al [22]: 32% of apps complied with item 12, and 36% of apps satisfied item 13; 23% and 27% complied with these items in our study. Finally, Papageorgiou et al [20] evaluated item 14: 58% of apps informed users about profiling. This result was quite different from ours: 9% satisfied item 14.

Like other privacy scales [18,19,27], our scale considers each item to be equally important. In further research, we will work on the next iteration of the scale, wherein this approach will be reconsidered. We will evaluate whether using weighted scores provides a better assessment of the privacy policies of mHealth apps or only makes the scale more complex without any additional benefit.

### Limitations

This study has some limitations. Some relevant apps may have been missed during our searches due to limitations of the Google Play search algorithm. Also, it is possible that developers may not have included some relevant information in the app description. As the eligibility assessment was based on app descriptions in the first search, this lack of information might have resulted in app exclusion. Only the Spanish version of the Google Play website was used during the search, and potentially relevant apps published on other versions of Google Play might have been excluded. Our study focused on Android apps, and this restriction also could have introduced a selection bias.

### Conclusions

In this paper, we presented an improved version of our GDPR-based scale for the assessment of the fairness of privacy policies of mHealth apps. This new version has been successfully applied in a case study where the privacy policies of 31 cancer apps were analyzed, yielding results in line with similar studies. This analysis uncovered a surprising lack of fairness in these policies. The nature of the data and the concerns that patients have regarding privacy suggest that it should be a major concern for developers, users, and data controllers. Thus, the proposed scale seems to be suitable for evaluating the fairness of mHealth app privacy policies and for use by developers to ensure compliance with the GDPR.

## Appendix

Multimedia Appendix 1 User's guide.

Multimedia Appendix 2 List of mHealth apps.

## Abbreviations

Cat1: category 1
Cat2: category 2
Cat3: category 3
Cat4: category 4
DPO: data protection officer
EU: European Union
GDPR: General Data Protection Regulation
MARS: Mobile Application Rating Scale
mHealth: mobile health
N/A: not applicable
NPP: no privacy policy
