# Supplementary material for: Assessment of the Fairness of Privacy Policies of Mobile Health Apps: Scale Development and Evaluation in Cancer Apps
Source: JMIR Mhealth Uhealth. 2020 Jul 28;8(7):e17134. doi: 10.2196/17134 (PMC7420637; doi:10.2196/17134)
Supplement: Multimedia Appendix 1 [file mhealth_v8i7e17134_app1.docx]

# Appendix 1. User’s guide

Appendix 1 presents an user’s guide that can be used by developers as a checklist to design privacy policies that comply with GDPR, by data controllers to check if their apps are GDPR-compliant, or by app users that are demanding with the privacy of the apps they are utilizing. When assessing a privacy policy with our scale, several items must be checked, leading to a score for a privacy policy. Some items are evaluated binarily, with 0 points or 1 point, but others may be evaluated with 0.5 points if the privacy policy is not clear enough - see details below-. Keep in mind that some items may be not applicable (N/A) under certain circumstances.

One of the principles of the General Data Protection Regulation (GDPR) is being transparent with the data subject, thus this assessment guide penalizes privacy policies that omit some information or are unclear. Table 1 shows a summary of the items and their possible scores. Meanwhile, Tables 2 to 15 show a detailed description of every item, how are they obtained, their possible scores, and real examples from mHealth apps’ privacy policies

Table 1. Summary of items of the privacy scale

| **Item #** | **Name** | **Description** | **0.5 points possible** | **N/A Possible** |
| --- | --- | --- | --- | --- |
| 1 | Identity of data controller | Identify (name) the data controller and give contact details (name, postal address and electronic address). | Yes | No |
| 2 | Identity of the representative | If data controller is not in the EU, the identity of the representative must be given. | No | Yes |
| 3 | Data Protection Officer (DPO) details | A way to contact Data Protection Officer must be given | No | No |
| 4 | Purposes for the processing | The purposes for the processing must be explicit. | Yes | No |
| 5 | Legal basis for the processing | GDPR sets six legal bases for the processing: consent, necessary to perform a contract, legal obligation, protect vital interest of somebody, public interest or exercise official authority, and legitimate interest. One or more of them must be explicit in the privacy policy. | No | No |
| 6 | Legitimate interests from controller | If legitimate interest is stated as basis for the processing, the privacy policy must include them. | No | Yes |
| 7 | Recipients (or categories of recipients) of the personal data | The recipients (or categories of recipients) of the personal data must be explicit in the privacy policy | No | No |
| 8 | Transfers to non-EU countries | This item refers to the fact that personal data may be transferred to a country not in the EU | Yes | Yes |
| 9 | Period for which data will be stored | Privacy policy must point out when data will be erased | Yes | No |
| 10 | Existence of data subject’s rights | User’s rights under GDPR are the following: right to access, rectification, erasure, restriction of processing, object of processing and data portability. These must be mentioned in the privacy policy and there must also be an explicit way to exercise them. | Yes | No |
| 11 | Existence of the right to withdraw consent | This is an additional right that only exists if the legal basis for the processing is consent. | No | No |
| 12 | Right to lodge a complaint with a supervisory authority  it | This right must be mentioned in the privacy policy and there must also be an explicit way to exercise | Yes | No |
| 13 | Obligation to provide personal data | The privacy policy must explicitly state what happens if the user does not provide certain personal data | No | No |
| 14 | Existence of automated decision-making or profiling | There must be a reference to the existence or not of automated decision-making of profiling based on personal data in privacy policy | Yes | No |

Table 2. Item 1. Identity of data controller

| **Item number:** 1 | | |
| --- | --- | --- |
| **Name:** Identity of data controller | | |
| **Short description:** Identify (name) the data controller and give contact details (name, postal address and electronic address). | | |
| **Source:** GDPR defines data controller as “*the natural or legal person, public authority, agency or other body which, alone or jointly with others, determines the purposes and means of the processing of personal data*”. Data controller identification must be explicit in the privacy policy. | | |
| **Score:** 1 point if full information is given. Full information means name, postal address, and electronic address (both email and a contact form are considered valid) of the data controller. 0.5 points if some information is missing. 0 points if the information is omitted. If only an electronic address is provided, the score is 0 points. Also, if the street address is not mentioned, the score is 0 points. | | |
| **Examples** | **1 point** | *Healthbit, Kajaine House 57-67 High Street, Edgware, Middlesex HA8 7DD, United Kingdom, or contact us by using the contact us facility on our Site*. [1] |
|  | **0.5 points** | *Titular: LYX UROLOGIA S.L.P. C.I.F.: B86819836 Domicilio social: C/ General Perón, 20, 28020 Madrid Teléfono: 915134950* [2] |
|  | **0 points** | *Access to Information; Contacting Us:* *Please feel free to contact us if you have any questions about our Privacy Policy or our information practices. You may contact us as follows: privacy@outcomes4me.com* [3] |

Table 3. Item 2. Identity of the representative.

| **Item number:** 2 | | |
| --- | --- | --- |
| **Name:** Identity of the representative | | |
| **Short description:** If data controller is not in the EU, the identity of the representative must be given. | | |
| **Source:** GDPR states that if data controller is not in the EU, the identity of the representative must be given.  The representative is a natural or legal person, established in the EU, that must be designated by the data controller if it/he/she is no in the EU. | | |
| **Score:** 1 point is given if full information is given (in the same way as with the data controller). 0 points otherwise. | | |
| **Examples** | **1 point** |  |
|  | **0 points** |  |

Table 4. Item 3. Data Protection Officer (DPO) details.

| **Item number:** 3 | | |
| --- | --- | --- |
| **Name:** Data Protection Officer (DPO) details | | |
| **Short description:** A way to contact Data Protection Officer must be given. | | |
| **Source:** We assume that a DPO must exist in any mHealth application. Keep in mind that DPO may be an employee of data controller or an external person. | | |
| **Score:** At least, an email address must be given to get 1 point. DPO must be a different person from the data controller, so the email address must also be different. Otherwise, 0 points are given. | | |
| **Examples** | **1 point** | *All correspondence with regards to privacy should be addressed to:The Privacy Officer CancerAid Pty Ltd Level 4, Chris O’Brien Lifehouse 119-143 Missenden Road, Camperdown NSW 2050, Australia Email: contact@canceraid.com.au You may contact the Privacy Officer by email in the first instance* [4] |
|  | **0 points** |  |

Table 5. Item 4. Purposes for the processing.

| **Item number:** 4 | | |
| --- | --- | --- |
| **Name:** Purposes for the processing | | |
| **Short description:** The purposes for the processing must be explicit. | | |
| **Source:** GDPR mandates that the purposes for the processing must be explicit, somehow, in the privacy policy. | | |
| **Score:** Sometimes this information is given but information is too generic. For example, “We collect this information for the purpose of providing our service” does not give any detail about why the data controller needs the personal data. In this case, the score for this item is 0.5 points. If purposes are provided explicitly, 1 point is given. If some purposes are explicit in the privacy policy but, at the same time, some wildcard such as “any other purpose…” score will be 0.5 points. If purposes are not mentioned, 0 points. | | |
| **Examples** | **1 point** | *Broadly speaking, we use personal information for purposes of administering our business activities, providing customer service and making available other products and services to our customers and prospective customers. Occasionally, we may also use the information we collect to notify you about new services and special offers we think you will find valuable.*[5] |
|  | **0.5 points** | *While using our Service, we may ask that you provide us with certain information that can be used to contact or identify you Personally identifiable information may include, but is not limited to, your email address, full name or other information (“Personal Information”). We collect this information for the purpose of providing our Service*[6] |
|  | **0 points** |  |

Table 6. Item 5. Legal basis for the processing.

| **Item number:** 5 | | |
| --- | --- | --- |
| **Name:** Legal basis for the processing | | |
| **Short description:** GDPR sets six legal bases for the processing. One or more of them must be explicit in the privacy policy. | | |
| **Source:** The six legal bases for the processing set by GDPR are: consent, necessary to perform a contract, legal obligation, protect vital interest of somebody, public interest or exercise official authority, and legitimate interest. This information must be given in the privacy policy. | | |
| **Score:** This information must be given in the privacy policy in order to get 1 point. However, some privacy policies give this information embedded in the declaration of the purposes for the processing. As the information is, after all, given to the data subject, score will be 1 point in these cases. Privacy policies that mention all legal bases, or privacy policies that, somehow, enumerate an excessive number of legal bases in an effort to have a catch-all will also get 0 points. Furthermore, if 0 points are given to this indicator, indicators based on the legal basis (i.e. items 6 and 11) are considered N/A (not applicable). | | |
| **Examples** | **1 point** | *By registering for and using the Service you consent to the transfer of information to the U.S. or to any other country in which the GRYT App or its Service Providers maintain facilities and the use and disclosure of information about you as described in this Privacy Policy***.** [7] |
|  | **0 points** |  |

Table 7. Item 6. Legitimate interests from controller

| **Item number:** 6 | | |
| --- | --- | --- |
| **Name:** Legitimate interests from controller | | |
| **Short description:** If legitimate interest is stated as basis for the processing, the privacy policy must include them. | | |
| **Source:** GDPR states that, when applicable, the legitimate interest of the data controller must be given. | | |
| **Score:** 1 point if this information is given, 0 points otherwise. N/A if legitimate interest is not stated as a legal basis for the processing or if item 5 is 0 points. | | |
| **Examples** | **1 point** | *We process your information for our legitimate interests while applying appropriate safeguards that protect your privacy. This means that we process your information for things like detecting, preventing, or otherwise addressing fraud, abuse, security, usability, functionality or technical issues with our services, protecting against harm to the rights, property or safety of our properties, or our users, or the public as required or permitted by law; Enforcing legal claims, including investigation of potential violations of this Privacy Policy; in order to comply and/or fulfil our obligation under applicable laws, regulation, guidelines, industry standards and contractual requirements, legal process, subpoena or governmental request, as well as our TOS* [8] |
|  | **0 points** |  |

Table 8. Item 7. Recipients (or categories of recipients) of the personal data.

| **Item number:** 7 | | |
| --- | --- | --- |
| **Name:** Recipients (or categories of recipients) of the personal data | | |
| **Short description:** The recipients (or categories of recipients) of the personal data must be explicit in the privacy policy. | | |
| **Source:** GDPR mandates that the recipients (or categories of recipients) of the personal data must be explicit in the privacy policy. | | |
| **Score:** 1 point if this information is given, 0 points otherwise. We must note that, if there are no recipients, it must be explicitly stated. Also, we must keep in mind that, in accordance with GDPR, the person or entity that process personal data on behalf of the data controller (i.e. the data processor) is considered a recipient. Also, it is not compulsory to specify the specific name of the recipient so, this means it is right to refer to a category of recipient. For example, it is valid to refer to “a cloud provider” as recipient without specifying the specific name of that provider. | | |
| **Examples** | **1 point** | *Service Providers; We may employ third-party companies and individuals due to the following reasons: To facilitate our Service; To provide the Service on our behalf; To perform Service-related services; or To assist us in analyzing how our Service is used. We want to inform users of this Service that these third parties have access to your Personal Information*. [9] |
|  | **0 points** |  |

Table 9. Item 8. Transfers to non-EU countries.

| **Item number:** 8 | | |
| --- | --- | --- |
| **Name:** Transfers to non-EU countries | | |
| **Short description:** This item refers to the fact that personal data may be transferred to a country not in the EU (in fact, EU plus Norway, Liechtenstein and Iceland) | | |
| **Source** This item refers to the fact that personal data may be transferred to a country not in the EU. In this case, the data controller must give enough information about the measures that are in place to achieve a similar level of protection. GDPR states that this information must be given if there are transfers to non-EU countries and says nothing if the data is stored within the EU. However, we consider that this information must be given even where are not any transfers to non-EU countries. | | |
| **Score:** We give 1 point if privacy policy indicates that this transfer is in place and a reference to the measures. We consider enough information a reference to the compliance with Privacy Shield or similar frameworks. We also give 1 point if the transfer is based on an adequacy decision from the Commission. This “adequacy decision” refers to a country that, by decision of the European Commission, is considered as “safe”, which means that no further safeguard is needed. See https://ec.europa.eu/info/law/law-topic/data-protection/international-dimension-data-protection/adequacy-decisions for further details and a country list. If there is a reference to a transfer to non-EU countries without further information, this item is 0.5 points. If there is no information about transfers outside the EU, this item is 0 points. When the data controller is inside the EU, the fact of not transferring data outside the EU must be explicitly stated and the item gets N/A. If not, the item is 0 points. | | |
| **Examples** | **1 point** | *The data we collect through the Site may be stored and processed in any country where we have facilities or in which we engage service providers, including in the U.S. and where our affiliates operate. Some non-EEA countries are recognized by the European Commission as providing an adequate level of data protection according to EEA standards (the full list of these countries is available here. For transfers from the EEA to countries not considered adequate by the European Commission, we have put in place adequate measures, such as by ensuring that the recipient is bound by EU Standard Contractual Clauses, to protect your Personal Data. You may obtain a copy of these measures by contacting us as indicated in the Contact Us section*. [10] |
|  | **0.5 points** | *This website is hosted in the State of Texas in the United States of America and is subject to Texas and U.S. federal law. Our offices are located in the State of New York. If You are accessing this website from other jurisdictions, please be advised that You are transferring Your personal information to us in Texas and New York, and by using any of this website, You consent to that transfer and to abide by the applicable laws of the States of Texas and New York and applicable U.S. federal law concerning Your use of the websites and Your agreements with us. Any persons accessing this website from any jurisdiction with laws or regulations governing the use of the Internet, including personal data collection, use and disclosure different from those of the jurisdictions mentioned above may only use the websites in a manner lawful in their jurisdiction. If Your use of this website would be unlawful in Your jurisdiction, please do not use this website*. [11] |
|  | **0 points** |  |

Table 10. Item 9. Period for which data will be stored.

| **Item number:** 9 | | |
| --- | --- | --- |
| **Name:** Period for which data will be stored | | |
| **Short description:** Privacy policy must point out when data will be erased. | | |
| **Source:** GDPR mandates that the privacy policy must point out when data will be erased. | | |
| **Score:** To obtain 1 point, privacy policy must point out a specific time in the future when data will be erased. We consider the following time references as valid: a period of inactivity in the data subject’s account or a specific reference to a user request to erase the data. The last one is independent from the specification of data subject’s rights in the privacy policy.0.5 points if there is a reference to data erasure but there is no reference to a time in the future when data will be deleted. 0 points if there is no reference at all about data erasure | | |
| **Examples** | **1 point** | *As a general rule, we will hold your information for a period of up to seven years from the end of your relationship with the charity in accordance with our data retention policy. In some circumstances, this will be shorter. For example, information related to unsuccessful job applications is destroyed after two years. In some circumstances, this will be longer, for example, pension information of former employees and information relating to the research grants we’ve made. If you would like to know how long we will hold any specific information, then please contact us and we can provide further details.* [12] |
|  | **0.5 points** | *We will retain your personal information for as long as you use the Services, and consistent with our data retention policies* [13] |
|  | **0 points** |  |

Table 11. Item 10. Existence of data subject’s rights.

| **Item number:** 10 | | |
| --- | --- | --- |
| **Name:** Existence of data subject’s rights | | |
| **Short description:** User’s rights under GDPR are the following: right to access, rectification, erasure, restriction of processing, object of processing and data portability. These must be mentioned in the privacy policy and there must also be an explicit way to exercise them. | | |
| **Source:** GDPR mandates that the privacy policy must point out the user’s rights. | | |
| **Score:** 1 point is given if the specific user’s rights mentioned in GDPR (right to access, rectification, erasure, restriction of processing, object of processing and data portability), are pointed out in the privacy policy and there is a method to exercise these rights (such as an email or postal address). This information may also be provided using a link. 1 point if five or more rights are mentioned, and 0.5 points if partial information is given (for example, some rights are omitted or there is no indication on how to exercise the rights). 0 points if there is no reference to data subject’s rights. | | |
| **Examples** | **1 point** | *You have the right to request from us access to your own personal information. This is sometimes known as a 'subject access request'.*  *You also have the right to ask us not to process your personal data for direct marketing purposes. We will tell you if we intend to use your data for this purpose or if we intend to disclose your information to any third party for this purpose. You can exercise your right to prevent such processing by checking certain boxes on the forms we use to collect your data or by contacting our data protection officer.*  *From 25 May 2018, you will have additional rights to request from us:*  *That any inaccurate information we hold about you is corrected*  *That your information is deleted*  *That we stop using your personal information for certain purposes*  *That your information is provided to you in a portable format*  *That decisions about you are not made by wholly automated means*  *Many of the rights listed above are limited to certain defined circumstances and we may not be able to comply with your request. We will tell you if this is the case.*  *If you choose to make a request to us, we will aim to respond to you within one month. We will not charge a fee for dealing with your request.*  *If you are dissatisfied with how we are using your personal information or if you wish to complain about how we have handled a request, then please contact our Data Protection Officer and we will try to resolve any issues you may have.* [14] |
|  | **0.5 points** | *If you are certain that you will never want to use your OWise account again you can request us to delete your account. This includes all the details that you have ever entered, including all information, photos and audio recordings. Once deleted this information will not be accessible anymore. Also, you have the right to have your data exported so that you may use this in another way. Please contact us at privacy@owise.uk to make these requests.* [15] |
|  | **0 points** |  |

Table 12. Item 11. Existence of the right to withdraw consent.

| **Item number:** 11 | | |
| --- | --- | --- |
| **Name:** Existence of the right to withdraw consent | | |
| **Short description:** This is an additional right that only exists if the legal basis for the processing is consent | | |
| **Source:** GDPR states that, when applicable, the existence of the right to withdraw consent must be explicit in the privacy policy. | | |
| **Score:**  One point if this right is mentioned, along with the way of exercising it, and 0 points if the right is omitted. We must keep in mind that this item will be N/A if consent is not one of the legal bases for the processing or if item 5 is 0 points. | | |
| **Examples** | **1 point** | *Withdrawal of consent: You can withdraw the consent that you provided when you signed up to join the MyBCTeam community by deleting your account here: https://www.mybcteam.com/users/account/edit If you withdraw consent you will be completely excluded from the MyBCTeam Community* [16] |
|  | **0 points** |  |

Table 13. Item 12. Right to lodge a complaint with a supervisory authority.

| **Item number:** 12 | | |
| --- | --- | --- |
| **Name:** Right to lodge a complaint with a supervisory authority | | |
| **Short description:** This right must be mentioned in the privacy policy and there must also be an explicit way to exercise it. | | |
| **Source:** According to the GDPR, there is the obligation to inform users that they have the right to lodge a complaint with a supervisory authority if they consider that their rights have been violated. | | |
| **Score:** To score 1 point, the privacy policy must not only identify the appropriate supervisory authority but provide at least a link to it. Also, 1 point is given if the policy links to the list of all supervisory authorities within the EU. Simply naming the supervisory authority is not considered enough and the score is 0.5 points. 0 points otherwise. | | |
| **Examples** | **1 point** | *You may lodge a complaint with a data protection authority competent for your country or region or place of alleged infringement. Please click here for contact information for such authorities* [10] |
|  | **0.5 points** | *You also have the right to lodge a complaint with a supervisory authority*. [8] |
|  | **0 points** |  |

Table 14. Item 13. Obligation to provide personal data.

| **Item number:** 13 | | |
| --- | --- | --- |
| **Name:** Obligation to provide personal data | | |
| **Short description:** The privacy policy must explicitly state what happens if the user does not provide certain personal data. | | |
| **Source:** According to the GDPR, data controller must state what happens it the user does not provide personal data. | | |
| **Score:** The privacy policy must explicitly state what happens if the user does not provide certain personal data. Examples of good practices are the following: “if you choose not to provide data, we may not be able to provide you those services.”, “In order to join (…) you must provide (…)”. If this information is given, 1 point. If no information is given, 0 points. | | |
| **Examples** | **1 point** | *In order to join the MyBCTeam community, you must provide your name, e-mail address, date of birth and role (patient, parent, or spouse). While participating in the community or otherwise using the Platform, you can also provide additional information such as your city, gender, profile image, diagnosis treatment information and other health- related information* [16] |
|  | **0 points** |  |

Table 15. Item 14. Existence of automated decision-making or profiling.

| **Item number:** 14 | | |
| --- | --- | --- |
| **Name:** Existence of automated decision-making or profiling | | |
| **Short description:** There must be a reference to the existence or not of automated decision-making of profiling based on personal data in privacy policy. | | |
| **Source:** We consider there must be a reference to the existence or not of automated decision-making of profiling based on personal data. This item may be tricky to interpret because sometimes applications make “automated decisions”, such as defining user interface language based on some personal data. This fact does not probably fit within the reasoning of the GDPR. Thus, we consider as automated decision-making or profiling a behavior that go further than simple “decisions” made by the app. | | |
| **Score:** This item scores 1 point if this information is shown in the privacy policy and enough information about the logic around this decision or profiling is given. We consider that a link to the information as valid. 0.5 points are scored if there is a reference to this item but with no additional information, and the score is 0 points if no information is given. Simply using Google Analytics is not enough to consider that the app is profiling. However, the use of cookies, if they modify the app behavior, is considered profiling. We also notice that, following recommendations from the Spanish supervisory authority on mobile apps, information must be given if the app includes targeted advertisements. | | |
| **Examples** | **1 point** | *Our Apps use automatic data collection technologies including first- and third- party Cookies, log files, Web Beacons, Pixels, dynamic tags, and other technical means. We may use these technologies to collect information about your online activities over time and across third-party websites or other online services in order to deliver content and advertising tailored to your interests, both on our Apps and on third-party applications (including but not limited to search engines and social media applications) as well as through our Website and other electronic communications. This practice is commonly known as Online Behavioral Advertising.*  *The third-parties who provide us with third-party Cookies, Web Beacons, Pixels, dynamic tags, and other tracking technologies, may use these technologies to collect information about you when you use the App in order to provide you with advertising based on your use of the App and to track performance of their advertising. Third-parties may have the ability to link the information they collect about you when you use the App with other information they collect about you elsewhere on the Internet, including but not limited to your PII or information about the mobile device you are using. Third- parties may also collect information about your online or application activities over time and across different websites, applications, and other online services and may use this information to provide you with Online Behavioral Advertising or other targeted content*. [17] |
|  | **0.5 points** | *MHT uses cookies to automatically gather, analyze, and store technical information about Platform visitors (e.g., visitor’s IP address, browser type, data about the pages visited on our Platform). This information is used to improve MHT’s service, as well as customize and enhance the experience of our Platform visitors.*  *For more information about the cookies used on our Platform, the third-party service providers that we use to gather such information, and reports that we receive based on the use of tracking technologies, please refer to our Cookies Policy at https://www.mybcteam.com/about/cookies_policy*  *You can control the use of cookies at the individual browser level, but if you choose to disable cookies, it will limit your use of certain features or functions on our Platform or service, affecting your Platform*  *experience. To manage cookies, please click here https://www.mybcteam.com/about/cookies_policy* [16] |
|  | **0 points** | *In certain circumstances we will use information about you from publicly available sources such as online registries, websites, media or social media, or personal introductions in order to understand more about your interests and preferences so that we can better tailor our communications – telling you about the things you are likely to be interested in, letting you know of ways to fundraise with us which are relevant to you and making sure that we only talk to you about a financial level of giving that is appropriate to you. We may do this by looking at your career information, peer networks, demographic information, hobbies and interests or other information.* [12] |

## References

[1] Pancreatic Cancer Action - symptom tracker. https://www.healthbit.com/privacy-policy/-. Last accessed: July 2019.

[2] Focalyx. http://www.focalyx.es/informacion-legal/. Last accessed: July 2019.

[3] Outcomes4me. https://www.outcomes4me.com/privacy. Last accessed: July 2019.

[4] Cancer AID. https://www.canceraid.com/confidentiality/. Last accessed: July 2019.

[5] inKind Space. http://intro.inkindspace.com/privacy-policy. Last accessed: July 2019.

[6] Boobytrap - The Breast Cancer App. https://www.boobytrapp.io/privacy-policy. Last accessed: July 2019.

[7] GRYT Health Cancer Community. https://app.grytapp.com/#/privacy-policy.html. Last accessed: July 2019.

[8] BELONG Beating Cancer Together. http://belong.life/privacy-policy/. Last accessed: July 2019.

[9] Cancer Surveillance. http://www.gomlv.com/privacy-policy.html. Last accessed: July 2019.]

[10] Emotion Space. https://privacycenter.pfizer.com/es/app/emotionspace. Last accessed: July 2019.

[11] Triple Negative Breast Cancer. https://kognito.com/privacy-policy/. Last accessed: July 2019.

[12] BECCA - Breast Cancer Support. https://www.breastcancercare.org.uk/privacy-cookies. Last accessed: July 2019.

[13] chemoWave: for cancer patients. http://chemowave.com/privacy/. Last accessed: July 2019.

[14] The BAPS App Wales. http://www.cardiffandvaleuhb.wales.nhs.uk/privacy-policy. Last accessed: July 2019.

[15] OWise breast cancer. http://www.owise.uk/privacy. Last accessed: July 2019.

[16] Breast Cancer Support. http://www.mybcteam.com/about/privacy. Last accessed: July 2019.

[17] Cancer.net Mobile. http://www.asco.org/about-asco/legal/privacy-policy. Last accessed: July 2019.
