# Supplementary material for: Assessment of the Fairness of Privacy Policies of Mobile Health Apps: Scale Development and Evaluation in Cancer Apps
Source: JMIR Mhealth Uhealth. 2020 Jul 28;8(7):e17134. doi: 10.2196/17134 (PMC7420637; doi:10.2196/17134)
Supplement: Multimedia Appendix 2 [file mhealth_v8i7e17134_app2.docx]

# APPENDIX 2: List of mHealth apps

## List of mHealth Apps found in searches

| 1 | Cáncer de mama TRATAMIENTO  /store/apps/details?id=com.andromo.dev761382.app889754 |
| --- | --- |
| 2 | Cáncer de mama - Síntomas y causas  /store/apps/details?id=com.itatvadev.cancerdemamasintomasycausas |
| 3 | Cáncer de mama (riesgo, síntomas, tratamiento)  /store/apps/details?id=breast.cancer.symptomsandawareness |
| 4 | MAMA – TelessaúdeRS  /store/apps/details?id=br.ufrgs.telessauders.mmg |
| 5 | DearMamma lucha contra el cáncer de mama  /store/apps/details?id=de.seracom.dearmamma |
| 6 | Marcos Cancer de Mama fondos de pantalla  /store/apps/details?id=com.photoframegratis.marcoscancerdemamabreastcancerpictureframes |
| 7 | TNM8 - Estadio de Cáncer de Mama  /store/apps/details?id=com.roche.latam.tnm8 |
| 8 | El cáncer de mama  /store/apps/details?id=com.Breast.Cancer.Awareness |
| 9 | Brexa - Breast cancer screening  /store/apps/details?id=com.vesna.cancerscreening |
| 10 | Escuela de Pacientes: Aula de Cáncer de Mama  /store/apps/details?id=com.salumedia.escueladepacientes.aulacm |
| 11 | Breast Cancer  /store/apps/details?id=io.kodular.amirasim1001.Breast_Cancer |
| 12 | Breast Cancer Symptoms & Workout Increase Size  /store/apps/details?id=com.massagedroid.creastcancer.workoutincrease |
| 13 | BECCA - Breast Cancer Support  /store/apps/details?id=com.yourcompany.becca |
| 14 | Cancro da Mama  /store/apps/details?id=pt.lpcc.cancromama |
| 15 | El cáncer de mama  /store/apps/details?id=com.All.About.Breast.Cancer |
| 16 | EmotionSpace cáncer de mama  /store/apps/details?id=com.pfizer.es.EmotionSpace |
| 17 | Breast Cancer: Information about breast cancer  /store/apps/details?id=com.doctorapps.breastcancer |
| 18 | cancer du sein  /store/apps/details?id=com.andromo.dev667101.app681429 |
| 19 | chemoWave: for cancer patients  /store/apps/details?id=com.chemowave.android |
| 20 | Breast Cancer Risk Assessment  /store/apps/details?id=com.mizSoftware.bcra |
| 21 | Touch Saúde  /store/apps/details?id=com.touchbrasil.touchsaude |
| 22 | Autoexamen de mamas  /store/apps/details?id=com.andromo.dev689019.app726582 |
| 23 | El cáncer de mama  /store/apps/details?id=tes.funnydivertisement.com.breast |
| 24 | Anti Breast Cancer Diet  /store/apps/details?id=com.andromo.dev677250.app828022 |
| 25 | Breast Cancer App ( breast cancer stages )  /store/apps/details?id=stages.breast.cancer |
| 26 | Autoexploración Mamaria  /store/apps/details?id=com.mobincube.android.sc_GS55Y |
| 27 | Breast Cancer Symptoms  /store/apps/details?id=breast.cancer.symptoms |
| 28 | OWise breast cancer  /store/apps/details?id=nl.onesixty.owise |
| 29 | My Cancer Coach  /store/apps/details?id=com.genomichealth.mycancercoach |
| 30 | Breast Examination  /store/apps/details?id=com.sana.nextgen.breastcancer.app |
| 31 | El cáncer de mama  /store/apps/details?id=com.andromo.dev474745.app474063 |
| 32 | Breast Cancer  /store/apps/details?id=com.andromo.dev663676.app666377 |
| 33 | Conciencia del cáncer de mama  /store/apps/details?id=com.Breast.Cancer.advice |
| 34 | Breast Advocate  /store/apps/details?id=com.breastadvocate.android |
| 35 | Breast Cancer Support  /store/apps/details?id=com.myhealthteams.MyBCTeam |
| 36 | KMBCN  /store/apps/details?id=org.kobura.kmbcn |
| 37 | Breast Cancer  /store/apps/details?id=com.andromo.dev565055.app665447 |
| 38 | Breast Cancer  /store/apps/details?id=com.magna.breastcancer |
| 39 | স্তন ক্যান্সার সচেতনতা Breast Cancer  /store/apps/details?id=com.ertapps.breast_cancer_new |
| 40 | Breast Cancer Survival Guide for Patients  /store/apps/details?id=com.andromo.dev658544.app972355 |
| 41 | Breast Cancer Symptoms  /store/apps/details?id=com.epicsol.breastcancer |
| 42 | Triple Negative Breast Cancer  /store/apps/details?id=com.Kognito.TNBC |
| 43 | Breast Cancer Awareness  /store/apps/details?id=com.breastcancer.ska |
| 44 | Breast Cancer Awareness SRIOR  /store/apps/details?id=com.magna.srior.breastcancer |
| 45 | Cancer de mama  /store/apps/details?id=com.mmi.breastcancer |
| 46 | Breast Cancer App ( breast cancer awareness )  /store/apps/details?id=awareness.breast.cancerr |
| 47 | Breast Cancer Kent Patient App  /store/apps/details?id=uk.org.breastcancerkent.bpkentoncology |
| 48 | Breast Cancer  /store/apps/details?id=com.andromo.dev737641.app807664 |
| 49 | Breast Cancer  /store/apps/details?id=com.andromo.dev547329.app794767 |
| 50 | Breast Cancer Initiative EA  /store/apps/details?id=com.bceastafrica.bciea |
| 51 | Breast Cancer Stages, Signs, Food and Meal Plan  /store/apps/details?id=breast.cancer.day.health.woman.mealplan |
| 52 | Breast Cancer Recovery  /store/apps/details?id=au.com.appliquette.breastcancerrecovery |
| 53 | BREAST CANCER SYMPTOMS  /store/apps/details?id=breast.cancer.symptoms.yai |
| 54 | Breast Cancer - Others Like Me  /store/apps/details?id=com.OthersLikeMe.BreastCancer |
| 55 | Breast Check Now  /store/apps/details?id=uk.org.breakthrough.ibreastcheck |
| 56 | Pandekha - Breast Cancer Self Examination  /store/apps/details?id=com.virtualtechmalawi.pandekha |
| 57 | Check Yourself!  /store/apps/details?id=org.keep_a_breast.keepabreast |
| 58 | Cáncer de garganta  /store/apps/details?id=com.andromo.dev474745.app516861 |
| 59 | El cáncer nasofaríngeo  /store/apps/details?id=com.andromo.dev474745.app553521 |
| 60 | Cáncer uterino  /store/apps/details?id=com.andromo.dev474745.app516865 |
| 61 | CheckMate Breast Cancer  /store/apps/details?id=com.multiplexer.checkmate |
| 62 | Ámate \| Cuida tu salud  /store/apps/details?id=org.pfccap.education |
| 63 | Breast Cancer Guide  /store/apps/details?id=com.droidmedic.breastcancer |
| 64 | Breas Cancer App ( metastatic breast cancer )  /store/apps/details?id=metastatic.breast.cancer |
| 65 | Cancer de RIÑON  /store/apps/details?id=com.andromo.dev474745.app542245 |
| 66 | Signs & Symptoms Breast Cancer  /store/apps/details?id=com.builtbydoctors.ssbreastcancer |
| 67 | Breast Cancer App ( symptoms of breast cancer )  /store/apps/details?id=signsandsymptomsof.breast.cancer |
| 68 | Cáncer de estómago  /store/apps/details?id=com.andromo.dev474745.app516856 |
| 69 | ClinTrial Refer Breast Cancer  /store/apps/details?id=com.lps.clintrialbreastcancer |
| 70 | Breast Cancer  /store/apps/details?id=com.codore.hypnosis_breast_cancer |
| 71 | 300 Tips to prevent Cancer  /store/apps/details?id=com.andromo.dev462136.app546291 |
| 72 | Alimentation Anti Cancer  /store/apps/details?id=com.andromo.dev522101.app497464 |
| 73 | Breast Cancer Symptoms  /store/apps/details?id=com.androiderapps.breastcancersymptoms |
| 74 | Breast Cancer Information  /store/apps/details?id=com.kimung.breastcancer |
| 75 | Breast Cancer  /store/apps/details?id=com.focusmedica.ud.breastcancer |
| 76 | BREAST CANCER ASSESSMENT  /store/apps/details?id=com.cipl.breastcancer |
| 77 | স্তন বা ব্রেস্ট ক্যান্সার - সমস্যা ও সমাধান  /store/apps/details?id=com.BreastCancer.health |
| 78 | 유방암 by 세컨드 닥터  /store/apps/details?id=com.mediplussolution.android.csmsrenewal.breastcancer |
| 79 | Breast Cancer Care  /store/apps/details?id=com.thereyv.breastcancercare |
| 80 | cáncer de ojo  /store/apps/details?id=com.andromo.dev474745.app553506 |
| 81 | Know Your Lemons  /store/apps/details?id=com.knowyourlemons.app |
| 82 | Breast Cancer Symptoms  /store/apps/details?id=com.droidmedic.breastcancersymptoms |
| 83 | B4BC Boarding 4 Breast Cancer  /store/apps/details?id=com.blacksunpro.b4bc |
| 84 | Cáncer de cuello uterino  /store/apps/details?id=com.andromo.dev474745.app475346 |
| 85 | Breast Cancer  /store/apps/details?id=com.incotradebusniesssolutionsp.breastcancer |
| 86 | El cáncer anal  /store/apps/details?id=com.andromo.dev474745.app553500 |
| 87 | BBCS 2019  /store/apps/details?id=br.com.conexaoproweb.appbbcs2019 |
| 88 | Outcomes4Me  /store/apps/details?id=com.outcomes4me |
| 89 | Breast cancer  /store/apps/details?id=com.andromo.dev576843.app593718 |
| 90 | Boobytrapp - The Breast Cancer App  /store/apps/details?id=com.boobytrapp |
| 91 | Brest Cancer App ( signs of breast cancer )  /store/apps/details?id=signsof.breast.cancer |
| 92 | El cáncer de vulva  /store/apps/details?id=com.andromo.dev474745.app553527 |
| 93 | Terbaru Ramuan Herbal Kanker Payudara  /store/apps/details?id=com.ramuanherbalkankerpayudara.kumpulanramuan |
| 94 | cáncer del apí©ndice  /store/apps/details?id=com.andromo.dev474745.app558317 |
| 95 | My Breast Cancer Advocate  /store/apps/details?id=com.patricia.cancerapp |
| 96 | Breast Cancer Awareness  /store/apps/details?id=com.jeannapps.breastcancerinformation |
| 97 | নারীদের স্তন ক্যান্সার~স্তনক্যান্সার থেকে মুক্তি  /store/apps/details?id=com.mjmtapps.breast_cancer |
| 98 | ক্যান্সার Cancer ~ রোগ ও চিকিৎসা  /store/apps/details?id=com.ayaatapps.cancer_info |
| 99 | El cáncer infantil  /store/apps/details?id=com.andromo.dev474745.app582011 |
| 100 | Breast Cancer Insights  /store/apps/details?id=com.shuru.kotlin.kotlin |
| 101 | El cáncer de paratiroides  /store/apps/details?id=com.andromo.dev474745.app553522 |
| 102 | Cancer de próstata  /store/apps/details?id=com.andromo.dev474745.app473200 |
| 103 | أعراض سرطان الثدي  /store/apps/details?id=comp.A3rad_Saratan_Tadiy |
| 104 | Know about CANCER Disease  /store/apps/details?id=com.andromo.dev542547.app881642 |
| 105 | Cáncer pancreático  /store/apps/details?id=com.andromo.dev474745.app474703 |
| 106 | Breast Centres Network  /store/apps/details?id=com.evtel.bcn.bcnappup |
| 107 | ক্যান্সার সচেতনতা~Cancer Awareness  /store/apps/details?id=com.noorapps.cancer_awareness |
| 108 | Breast anatomy in 3D  /store/apps/details?id=reapps.BreastAnatomy |
| 109 | স্তন ক্যান্সার (ছেলে ও মেয়ে ) \| Breast Cancer  /store/apps/details?id=com.breast.cancer.solution.tips |
| 110 | السرطان وطرق علاجه  /store/apps/details?id=join.konbrand.Cancer2 |
| 111 | El cáncer de pene  /store/apps/details?id=com.andromo.dev474745.app553523 |
| 112 | ONCOassist  /store/apps/details?id=com.oncoassist.core |
| 113 | The BAPS App Wales  /store/apps/details?id=com.velindre.baps |
| 114 | Breast Cancer  /store/apps/details?id=com.bc.dbp |
| 115 | cáncer de boca  /store/apps/details?id=com.andromo.dev474745.app516864 |
| 116 | Breast Cancer Awareness  /store/apps/details?id=com.tadapps.breast.cancer.awareness |
| 117 | Breast Cancer Symptoms  /store/apps/details?id=com.andromo.dev592442.app881404 |
| 118 | Tipos de Cáncer  /store/apps/details?id=com.andromo.dev657817.app774587 |
| 119 | TNM Cancer Staging Calculator  /store/apps/details?id=com.canxer.canxer |
| 120 | ক্যান্সার \| cancer  /store/apps/details?id=com.canser.problem.solution.tips |
| 121 | BELONG Beating Cancer Together  /store/apps/details?id=com.belongtail.belong |
| 122 | Cancer  /store/apps/details?id=cancer.com.blogspot.codedeveloped.cancer |
| 123 | Beat That Cancer  /store/apps/details?id=appinventor.ai_beatthatcancer12.Cancer |
| 124 | Breast Cancer Staging  /store/apps/details?id=com.canxer.breastcancer |
| 125 | Breast Cancer - News/Videos  /store/apps/details?id=com.buzzato.breastcancer |
| 126 | ABC OF BREAST HEALTH  /store/apps/details?id=devatech.kims |
| 127 | Breast Cancer  /store/apps/details?id=com.andromo.dev565044.app613786 |
| 128 | Cáncer de hí­gado  /store/apps/details?id=com.andromo.dev474745.app516862 |
| 129 | cáncer de glándula salival  /store/apps/details?id=com.andromo.dev474745.app578358 |
| 130 | Bolest nije kraj - Udruzenje (karcinom dojke)  /store/apps/details?id=com.bolestnijekraj |
| 131 | Breast and Pec Check  /store/apps/details?id=com.RosslynHill.BreastandPecCheck |
| 132 | Mammarosa borstkanker info.  /store/apps/details?id=com.codingdutchmen.android.mammarosa |
| 133 | Cancer du sein  /store/apps/details?id=com.hugge.elipscds.fr |
| 134 | Breast Cancer  /store/apps/details?id=com.bmooble.android.app.breastCancer |
| 135 | cáncer de vesí­cula biliar  /store/apps/details?id=com.andromo.dev474745.app553518 |
| 136 | علاج سرطان الثدي  /store/apps/details?id=tady.saratan.ilaj.com |
| 137 | Cáncer de pulmón  /store/apps/details?id=com.andromo.dev522931.app852935 |
| 138 | Breast Aware  /store/apps/details?id=com.breastcancer.ireland2 |
| 139 | 핑크아바타  /store/apps/details?id=com.snubi.healthavatar |
| 140 | RosaApp  /store/apps/details?id=br.com.rosa.app |
| 141 | Cáncer de vejiga  /store/apps/details?id=com.andromo.dev474745.app516860 |
| 142 | CuidAPPlas  /store/apps/details?id=fecma.addis.es.fecma |
| 144 | Bioconsciencia â€“ Mama  /store/apps/details?id=org.juancayuela.bioconsciencia.mama |
| 145 | PinkDrive  /store/apps/details?id=za.co.diyapp.console.android5c4accc5cb6bc |
| 146 | Cancer testicular  /store/apps/details?id=com.andromo.dev474745.app516857 |
| 147 | The Magic Tree  /store/apps/details?id=com.rayd.magictree |
| 148 | My Breast Friend  /store/apps/details?id=com.appsotutely.mybreastfriend |
| 149 | 命の食事 ナグモクリニック  /store/apps/details?id=jp.tenplus.pushapp.nagumo |
| 150 | Diana  /store/apps/details?id=com.roche.diana |
| 151 | Pink Knights  /store/apps/details?id=com.zulekha.alexis.meridian.pinkitnow |
| 152 | Reconstructive Breast Surgery and Oncoplastic  /store/apps/details?id=com.andromo.dev658544.app972354 |
| 153 | টিউমার রোগের চিকিৎসা ~ Tumor Treatment  /store/apps/details?id=com.Medical.Disease.Treatment.Tumor |
| 154 | Sensifemme guante de autoexploración mamaria  /store/apps/details?id=com.kavishapharma.sensifemme |
| 155 | Pink Caravan  /store/apps/details?id=com.pinkcaravan.mobileapp |
| 156 | Tratamiento para todas las enfermedades  /store/apps/details?id=com.appsgames.medicalskindiseases |
| 157 | breast cancer  /store/apps/details?id=com.andromo.dev670419.app685599 |
| 158 | Centrum Chorób Piersi UCK  /store/apps/details?id=pl.centrumchorobpiersi |
| 159 | লিভার কান্সার চিকিত্‍সা ~ Liver cancer Treatment  /store/apps/details?id=com.Medical.Disease.Treatment.Liver_cancer |
| 160 | স্তন ক্যান্সারের কারন-প্রতিকার  /store/apps/details?id=com.boishakhiapps.SthonCanchererKaron |
| 161 | Komen Italia  /store/apps/details?id=com.weareorigami.komen |
| 162 | Ramuan Herbal Kanker Payudara  /store/apps/details?id=com.ramuanmujarabherbal.ramuanherbalkankerpayudara |
| 163 | Clinical Cancer Journals  /store/apps/details?id=com.elsevier.stmj.jat.newsstand.cllc |
| 164 | PITA PINK  /store/apps/details?id=com.app.ykpi.projectpitapink |
| 165 | 《醫藥人》第182期  /store/apps/details?id=com.medcom.yiyaoren182 |
| 166 | GO Contacts EX Breast Cancer  /store/apps/details?id=com.beanstorm.gocontact.theme.bcancer |
| 167 | PA Breast Cancer Coalition  /store/apps/details?id=com.pabreastcancer.pbcc |
| 168 | AGO Mammakarzinom Empfehlungen  /store/apps/details?id=com.ago.guidelines |
| 169 | Cáncer de sangre  /store/apps/details?id=com.andromo.dev474745.app504241 |
| 170 | Mammakarzinom onkowissen  /store/apps/details?id=de.onkowissen.mbctransparent |
| 171 | Breast cancer  /store/apps/details?id=com.referencehunt.breastcancer |
| 172 | BreastSentiTTL  /store/apps/details?id=net.vhebron.breastsentittl |
| 173 | Cancer Therapy Advisor  /store/apps/details?id=com.usbmis.troposphere.chemad |
| 174 | PreveR Colo/Mama  /store/apps/details?id=com.ufpi.mayron.pc3 |
| 175 | SABCS  /store/apps/details?id=com.coreapps.android.followme.sabcs2016 |
| 176 | Check Melanoma  /store/apps/details?id=com.era.checkmelanoma |
| 177 | Choosing Value  /store/apps/details?id=edu.mcw.chosingWisely |
| 178 | Ruce na prsa  /store/apps/details?id=cz.rucenaprsa.android |
| 179 | CMMC  /store/apps/details?id=com.conduit.app_28e88a10b2994913804168b4e515ad86.app |
| 180 | Breast Cancer : Info and Cure  /store/apps/details?id=com.biemultimedia.Breastcancerinfo |
| 181 | Self-Check BRC  /store/apps/details?id=com.map2heal.selfcheck |
| 182 | طبيب العائلة ـ علاج السرطان  /store/apps/details?id=km.app.FamilyDoctorCancer |
| 183 | Mamocare  /store/apps/details?id=com.sjk.mamocare |
| 184 | Breast Cancer Canada  /store/apps/details?id=com.tobh.android.bcc_2018 |
| 185 | 2019 BCT Annual Scientific Mtg  /store/apps/details?id=mobile.appksVM6M5zDq |
| 186 | íšnicas y Valientes  /store/apps/details?id=com.cuplesoft.unicas |
| 187 | BREAST TEST  /store/apps/details?id=com.positiva.breasttestapp |
| 188 | Pinky Promise-Al Noor  /store/apps/details?id=com.alnoor.pinkpromise |
| 189 | Complete Breast Care  /store/apps/details?id=com.andromo.dev698216.app741709 |
| 190 | How to Measure Bra Size  /store/apps/details?id=com.brasizemeasure.ska |
| 191 | El cáncer oral  /store/apps/details?id=com.andromo.dev474745.app534315 |
| 192 | 19oct, Share Your Courage  /store/apps/details?id=es.diox.android.DWW_19oct |
| 193 | স্তনের রোগসমূহ ও আকর্ষণীয় করার উপায়~ Breast Cancer  /store/apps/details?id=com.perfect.breast.medical.tips |
| 194 | Vik Sein  /store/apps/details?id=fr.vik |
| 195 | Got Boobs?  /store/apps/details?id=com.app.gotboobs |
| 196 | Breast Health  /store/apps/details?id=mk.acibademsistina |
| 197 | BreastCancerGib  /store/apps/details?id=com.pereiratech.breastcancergib |
| 198 | Cáncer intestinal  /store/apps/details?id=com.andromo.dev474745.app516859 |
| 199 | 19oct(Only Gallery), Share you  /store/apps/details?id=es.diox.android.dww19gallery |
| 200 | Breast Awareness App  /store/apps/details?id=com.silkprousa.breastawarenessapp |
| 201 | Cervical Cancer - News/Videos  /store/apps/details?id=com.buzzato.cervicalcancer |
| 202 | Baheya  /store/apps/details?id=org.baheya.hospital |
| 203 | Alimentos contra el cancer  /store/apps/details?id=com.sandroparoi.anticancerfood |
| 204 | KNMSP  /store/apps/details?id=com.uigtc.MOH.KNMSP |
| 205 | iBE Connect  /store/apps/details?id=uelifesciences.ibreastexam |
| 206 | স্বাস্থ্য Health  /store/apps/details?id=com.anantaroy.health |
| 207 | DINI  /store/apps/details?id=com.asdev.dini |
| 208 | Breast cancer recurrence  /store/apps/details?id=dfolgado.predictor.breastcancerrecurrence |
| 209 | Omakuu  /store/apps/details?id=fi.eatech.omakuu |
| 210 | Best Ways To Prevent Breast Cancer  /store/apps/details?id=com.andromo.dev673306.app680506 |
| 211 | Rosa de Prevención  /store/apps/details?id=appinventor.ai_pgrilli17.finalverdeF |
| 212 | iSeno  /store/apps/details?id=com.daiuto.app |
| 213 | Breast Cancer Awareness CM 12  /store/apps/details?id=com.donate.breastcancer.theme |
| 214 | Nachsorge-App fí¼r Brustkrebs  /store/apps/details?id=de.osp_stuttgart.app |
| 215 | Thrive  /store/apps/details?id=com.emocha.thrive |
| 216 | Vik Breast  /store/apps/details?id=ai.vik.breast |
| 217 | Cancer Fighting Foods  /store/apps/details?id=cancerfightingfoods.www.cancerfightingfoods |
| 218 | Jet Pink  /store/apps/details?id=com.sor.jetpink |
| 219 | Cancer Cafe  /store/apps/details?id=org.cancer_cafe.muthoniMate |
| 220 | Braster Care  /store/apps/details?id=eu.braster.brasterandroid |
| 221 | PAPI WILO \|\| MUSICA MP3 2019  /store/apps/details?id=com.projectBUL.PWMUSICAMP32019 |
| 222 | The IBC Network  /store/apps/details?id=com.ibcnetwork.theibcnetwork |
| 223 | Грудь вперёд  /store/apps/details?id=app.rakgrudi.org |
| 224 | Telerradiologia Educativa  /store/apps/details?id=mx.shirooba.appteleradiologiaedu |
| 225 | Cancer Preventing Food  /store/apps/details?id=com.andromo.dev522101.app497462 |
| 226 | Cancer Risk Calculator  /store/apps/details?id=be.tdf_it.cancerrisk |
| 227 | INFOFISIO (Cí¢ncer de Mama)  /store/apps/details?id=com.infofisio.cancerdemama |
| 228 | Breast cancer mortality  /store/apps/details?id=dfolgado.predictor.breastcancermortality |
| 229 | FUNdraising  /store/apps/details?id=org.cancer.fundraising |
| 230 | Prevención Del Cáncer - FAQ  /store/apps/details?id=com.proyectoultra6 |
| 231 | Mastologia: do diagnóstico ao tratamento  /store/apps/details?id=com.sbm.ebook |
| 232 | Viviendo con Cáncer - FAQ  /store/apps/details?id=com.proyectoultra7 |
| 233 | mShakti by Indian Cancer Society  /store/apps/details?id=com.ics.nfxdigital_ip.icsapp |
| 234 | BGICC  /store/apps/details?id=com.bgicc.app |
| 235 | Pad Aware  /store/apps/details?id=com.tejuinomx.aware |
| 236 | Cancer Predictor  /store/apps/details?id=com.datakeenbd.cancerpredictor |
| 237 | Mamma Mia! Die Krebsmagazine  /store/apps/details?id=com.pressmatrix.mammamia |
| 238 | Breast Care: Helpful Tips To Keep Breasts Healthy  /store/apps/details?id=com.andromo.dev658544.app972360 |
| 239 | 2 Cali Breast Meeting  /store/apps/details?id=com.cbm2.app |
| 240 | Ferramenta de Avaliação de Risco de Câncer de Mama  /store/apps/details?id=com.farcm |
| 241 | KarmApp  /store/apps/details?id=com.ionicframework.karmapp915585 |
| 242 | inKind Space  /store/apps/details?id=io.pixeledge.inkind |
| 243 | cancer 101  /store/apps/details?id=sk.cancer |
| 244 | flacidez de mama  /store/apps/details?id=com.Sagging.Breasts |
| 245 | ক্যান্সার প্রতিরোধের খাবারসমূহ  /store/apps/details?id=com.itjogot.cancerpreventionmeal |
| 246 | Rosae Informa  /store/apps/details?id=es.bandomovil.rosae.informa |
| 247 | Dr Bhavana Parikh  /store/apps/details?id=com.hospital.drbhavanaparikh |
| 248 | CANEX Quality Of Life  /store/apps/details?id=com.ejercicio.ejercicio |
| 249 | Lung Cancer Risk Predictor  /store/apps/details?id=appinventor.ai_pavel_chtch.Tammegagi |
| 250 | Cancer Surveillance  /store/apps/details?id=com.gomlv.cancersurveillance |
| 251 | Rotterdam Prostate Cancer Risk  /store/apps/details?id=com.rpcrc |
| 252 | Prostate Cancer  /store/apps/details?id=com.andromo.dev712859.app826281 |
| 254 | Prostate cancer  /store/apps/details?id=com.focusmedica.ud.prostatecancer |
| 255 | Prevención cáncer de próstata  /store/apps/details?id=com.andromo.dev761382.app890337 |
| 256 | Prostate Cancer Calculator  /store/apps/details?id=com.borinfer.test |
| 257 | Prostate Cancer Guideline  /store/apps/details?id=com.URODevelopments.ProstateCancerGuideline |
| 258 | itsaMANTHING - Prostate Cancer  /store/apps/details?id=uk.org.prostaid |
| 259 | Prostate Cancer Treatment  /store/apps/details?id=prostate.cancer.treatment |
| 260 | Prostate Cancer  /store/apps/details?id=com.blogspot.codedeveloped.prostatecancer |
| 261 | Prostate Cancer Disease  /store/apps/details?id=com.kimung.prostatecancer |
| 262 | Recognize Prostate Cancer Symptoms  /store/apps/details?id=com.recognize.prostate.cancer.symptoms.achtech007 |
| 263 | IPCRC (Prostate Ca Calculator)  /store/apps/details?id=ipcrc.com.prostateriskcalculator |
| 265 | Comment prévenir le cancer de la prostate  /store/apps/details?id=com.ElisaDev.CommentEviterCancerDeLaProstate |
| 266 | Comment Eviter le Cancer de la Prostate - Cancer  /store/apps/details?id=com.Andykab.CancerDeProstate |
| 267 | Improve Prostate Health  /store/apps/details?id=com.andromo.dev516013.app761347 |
| 268 | Prostate Cancer - News/Videos  /store/apps/details?id=com.buzzato.prostate |
| 269 | Prostate In Focus  /store/apps/details?id=com.magna.glenmark.prostateinfocus |
| 270 | Partin Tables  /store/apps/details?id=com.imedicalapps.partintables |
| 271 | Prostateâ£Check  /store/apps/details?id=ch.prostatakrebs.ProstateCheck |
| 272 | Desinflama la Próstata  /store/apps/details?id=com.annge.desinflamaprostata |
| 274 | CPC Risk Calculator  /store/apps/details?id=dk.daman.cpc |
| 275 | 전립선암 계산기  /store/apps/details?id=org.snubh.prostate_calc |
| 277 | Comida contra el cáncer  /store/apps/details?id=com.chronosstudio.CancerFightingFood |
| 278 | Prostate Cancer  /store/apps/details?id=com.bmooble.android.app.prostateCancer |
| 280 | Prostate Cancer Conference 16  /store/apps/details?id=me.doubledutch.jvcdf.prostatecancerconference16 |
| 282 | Master Class en Cáncer de Próstata IVO  /store/apps/details?id=es.infobox.eventos.appMasterClassCaP |
| 283 | Support a Spouse With Prostate Cancer  /store/apps/details?id=com.support.aspouse.with.prostate.cancer.achtech007 |
| 284 | Como Curar La Próstata - Remedios Naturales Gratis  /store/apps/details?id=com.luzenlaoscuridad.comocurarlaprostataremediosnaturalesgratis |
| 287 | Uroonkologia  /store/apps/details?id=pl.com.ex.atlas.pub117 |
| 288 | Prostate Volume and Density  /store/apps/details?id=com.imedicalapps.prostatevolumeanddensity |
| 291 | 전립선암 by 세컨드 닥터  /store/apps/details?id=com.mediplussolution.android.csmsrenewal.prostatecancer |
| 294 | APPdrogeno  /store/apps/details?id=com.janssen.android.AndrogenoApp |
| 297 | Men's App - men's health  /store/apps/details?id=com.mensapp.en |
| 298 | Movember  /store/apps/details?id=com.movember.android.app |
| 299 | AdMeTech 2017 Global Summit  /store/apps/details?id=com.coreapps.android.followme.admetech17 |
| 300 | Focalyx  /store/apps/details?id=com.teravision.focalyx.android |
| 302 | Prevención de enfermedad  /store/apps/details?id=com.pulsar.brunotrstenjak.mdss_pro |
| 303 | Move and fight Cancer! - MetTracker  /store/apps/details?id=de.lindenvalley.mettracker |
| 304 | علاج البروستاتا  /store/apps/details?id=comp.Kayfiyat_3ilaj_Prostata |
| 305 | PP - Prostate Prognostic  /store/apps/details?id=appinventor.ai_coutobraulio.PP_Prostate_Prognostic |
| 306 | 3 Cara untuk Menjaga Kesehatan Prostat  /store/apps/details?id=com.tigacarauntukmenjagakesehatanprostat.anekatipskesehatandankecantikan |
| 307 | beneficios de la Saw Palmetto  /store/apps/details?id=com.fredshrodent.beneficiosdelasawpalmetto |
| 309 | Know Your Score WA  /store/apps/details?id=com.communitytogo.knowyourscorewa |
| 310 | PROSTATE INTERNATIONAL  /store/apps/details?id=com.m2comm.prostate.journal |
| 311 | SCaP calculator  /store/apps/details?id=ac.yuhs.scapcalculator.scap_calculator |
| 313 | Cáncer de ovarios  /store/apps/details?id=com.andromo.dev474745.app492780 |
| 314 | Benign Prostatic Hyperplasia Advice  /store/apps/details?id=benign.prostatic.hyperplasia.bph |
| 315 | Como Curar La Prostata - Remedios naturales Gratis  /store/apps/details?id=com.movilesapp.comocurarlaprostata |
| 317 | Adrenal Cancer - Others Like Me.  /store/apps/details?id=com.SameAsIAcc |
| 319 | How Are You Today? PC  /store/apps/details?id=com.intelesant.pc |
| 320 | طبيب العائلة ـ علاج السرطان  /store/apps/details?id=km.app.FamilyDoctorCancer |
| 321 | 2019 ANZUP ASM  /store/apps/details?id=au.org.anzup.the2019anzupasm |
| 322 | Cáncer colonrectal  /store/apps/details?id=com.andromo.dev474745.app485130 |
| 323 | علاج سرطان الثدي  /store/apps/details?id=tady.saratan.ilaj.com |
| 324 | ProsCApp  /store/apps/details?id=org.inspirecenter.eons |
| 325 | expected PSA&TIME in PCa-ADT  /store/apps/details?id=com.akbay.expectedpsatime |
| 326 | Men's App - Salud del hombre  /store/apps/details?id=com.mensapp.es |
| 327 | PCF Retreat  /store/apps/details?id=com.ocs.confpal.pcf |
| 328 | Prostatafibel  /store/apps/details?id=de.medizinwelten.prostatafibel |
| 329 | mCRPC Master  /store/apps/details?id=mCRPC.Droid |
| 331 | Todo Sobre Su Próstata  /store/apps/details?id=com.andromo.dev730669.app834577 |
| 332 | AdMeTech 18  /store/apps/details?id=mobile.app5XeTOY3hHU |
| 333 | Easy Ways to Treat Cancer  /store/apps/details?id=com.t_999appsdeveloper.Easy_Ways_to_Treat_Cancer |
| 334 | Cáncer de pulmón sintomas causas y tratamiento  /store/apps/details?id=com.itatvadev.cancerdepulmonsintomascausasytratamiento |
| 335 | A to Z Diseases with Causes and Treatment by 999  /store/apps/details?id=com.t_999appsdeveloper.Everyday_Health_Health_A_to_Z |
| 337 | That Dragon, Cancer  /store/apps/details?id=com.numinousgames.ThatDragonCancer |
| 338 | myProHealth  /store/apps/details?id=com.partners.myprohealth |
| 339 | Remedios para Próstata  /store/apps/details?id=com.andromo.dev605364.app598926 |
| 340 | IX Simposio Cientí­fico SOGUG  /store/apps/details?id=es.tactics.sogug2018 |
| 341 | EliFIT  /store/apps/details?id=com.physitrack.physiapp.elifit |
| 342 | Cancer Ko Kese Mitaye - Tips for Cancer Daises  /store/apps/details?id=com.special.healthtips.cancerhealthtips |
| 343 | Cancer 101 by GoLearningBus  /store/apps/details?id=com.quizmine.cancerandroid |
| 344 | 100% Cancer Cure & Prevention  /store/apps/details?id=daily.a.cancerpreventionandcure |
| 345 | F RECAPRO  /store/apps/details?id=com.pigskin.medicaltables.medicaltable |
| 346 | Справочник анализов - анализ крови, анализ мочи  /store/apps/details?id=com.codename26.childanalysis |
| 347 | Remedios Caseros  /store/apps/details?id=com.powerapps.remedioscaseros |
| 348 | Applied 40 herb for 100 health condition  /store/apps/details?id=com.t_999appsdeveloper.Applied_40_herb_for_100_health_condition |
| 349 | Prevención de cáncer  /store/apps/details?id=com.LeonTechnologies.CancerPreventionAntioxidants |
| 350 | TNM Cancer Staging  /store/apps/details?id=com.kkltd.tnm |
| 351 | Talk Genetics- Oncology  /store/apps/details?id=com.q7862cce4055 |
| 352 | patientMpower  /store/apps/details?id=com.maithu.transplantbuddy |
| 353 | Cancer Flashcards Ultimate  /store/apps/details?id=com.ablet.cancerflashcardsultimate |
| 354 | RT Tools  /store/apps/details?id=appinventor.ai_a_abdelkar.RTtools |
| 355 | Tablet Flashcards Cancer  /store/apps/details?id=com.ablet.tabletflashcardscancer |
| 356 | ClinTrial Refer ANZUP  /store/apps/details?id=com.lps.anzup |
| 357 | AINU  /store/apps/details?id=com.ainuindia.ainu |
| 359 | New Kegel Exercise - Prostate Exercise  /store/apps/details?id=com.eng.kegelise |
| 360 | Simposio Revisiones Cáncer 19  /store/apps/details?id=com.grupoaran.revcancer2019 |
| 361 | 新ケーゲル体操 - 前立腺トレーニング  /store/apps/details?id=com.jpn.kegelise |
| 363 | EAU Guidelines  /store/apps/details?id=org.european_association_urology.pocket_guidelines |
| 364 | DreamLab  /store/apps/details?id=au.com.vodafone.dreamlabapp |
| 366 | PillsBills â€“ Online Pharmacy  /store/apps/details?id=com.pillsbillsofficial.app |
| 367 | Symptoms and Treatment of top 40 diseases  /store/apps/details?id=com.t_999appsdeveloper.Symptoms_and_Treatment_of_Top_40_diseases |
| 368 | Oddway International  /store/apps/details?id=com.oddwayinternational.app |
| 369 | 뉴케겔운동 - 전립선운동  /store/apps/details?id=com.kegelise |
| 370 | Beneficios de la cebolla  /store/apps/details?id=com.LeonTechnologies.HealthBenefitsOfOnion |
| 371 | Health benefits of Cottonseed oil  /store/apps/details?id=mmdigitalproducts.com.healthbenefitCottonseedoil |
| 372 | iURO Oncology Pro  /store/apps/details?id=com.ACSimulation.iURO_OncoPro |
| 374 | 新凯格尔运动 - 前列腺运动  /store/apps/details?id=com.chi.kegelise |
| 375 | Cancer.Net Mobile  /store/apps/details?id=com.fueled.cancernet |
| 376 | Treatment of diseases by 999 Health Doctors  /store/apps/details?id=com.t_999appsdeveloper.Treatment_of_diseases_by_999_Health_Doctors |
| 377 | Alimentation Anti Cancer  /store/apps/details?id=com.andromo.dev518100.app479474 |
| 378 | Ramuan Alami Untuk Penyakit Prostat Paling Ampuh  /store/apps/details?id=com.ramuanalamiuntukpenyakitprostat.carinsurancefree |
| 380 | Nursing Cancer Deluxe  /store/apps/details?id=com.efn.nursingcancerdeluxe |
| 382 | beneficios de la Brócoli  /store/apps/details?id=com.fredshrodent.beneficiosdelabrocoli |
| 383 | Gurumann Info  /store/apps/details?id=com.wGurumanninfo_7824210 |
| 384 | TODAS las enfermedades de la sangre  /store/apps/details?id=com.Treatmento.bloodo |
| 385 | Chronic Disease  /store/apps/details?id=com.rjp.chronicdisease |
| 386 | Cura Milagrosa Para El Cáncer  /store/apps/details?id=com.edwarlugo.curamilagrosaparaelcancer |
| 387 | Cáncer de colon  /store/apps/details?id=com.andromo.dev522931.app852929 |
| 388 | Radiology Tutor  /store/apps/details?id=com.radiologytutor.calculators |
| 389 | Cancro Colorretal  /store/apps/details?id=pt.lpcc.cancrocoloretal |
| 390 | Nursing Cancer  /store/apps/details?id=com.efn.nursingcancer |
| 391 | Preguntas Sobre La Próstata  /store/apps/details?id=com.andromo.dev730669.app835052 |
| 392 | Medical terms Dictionary 2019  /store/apps/details?id=com.SF.medicaldictionary |
| 393 | Dr Abhay Jha  /store/apps/details?id=drabhay.drabhay |
| 394 | PatientsEngage Health Network  /store/apps/details?id=com.PatientsEngage.UI |
| 395 | Patient Education Institute  /store/apps/details?id=pl.com.ex.pei.atlas |
| 397 | Alcaraz Option  /store/apps/details?id=com.sfy.dralcaraz |
| 398 | ESMO Cancer Guidelines  /store/apps/details?id=com.appyzz.android.esmo |
| 400 | Chronic Disease And Treatment  /store/apps/details?id=com.app1daily.chronicdisease |
| 401 | TNM Cancer Staging  /store/apps/details?id=com.app.tnm |
| 402 | Mushroom Benefits  /store/apps/details?id=com.healthinfo.mushroom.benefits.weightloss.hairloss.skin.nutrition |
| 403 | Health benefits of Parsley  /store/apps/details?id=mmdigitalproducts.com.healthbenefitsofparsley |
| 404 | Ramuan Kanker Usus Paling Ampuh Mudah Di Dapat  /store/apps/details?id=com.ramuanherbalkankerlengkap.obatherbal.kumpulanramuanherbal.mesotheliaomalawfirm |
| 405 | Recetas de pescado  /store/apps/details?id=com.cooking.fishrecipes |
| 406 | Cancer du col utí©rin  /store/apps/details?id=com.andromo.dev667101.app686769 |
| 407 | Briganti Nomogram  /store/apps/details?id=bisbjerg.no_iDevice.briganti |
| 408 | Cara Alami Mengatasi Telinga Tersumbat Ampuh  /store/apps/details?id=com.obatalamimengatasitelingabudeklengkap.obatherbal.kumpulanramuanherbal.mesotheliaomalawfirm |
| 409 | Saw Palmetto  /store/apps/details?id=com.blogspot.codedeveloped.sawpalmetto |
| 410 | Cara Alami Mengobati Hidung Tersumbat Pada Bayi  /store/apps/details?id=com.caraalamimengobatihidungtersumbatpadabayilengkap.obatherbal.kumpulanramuanherbal.mesotheliaomalawfirm |
| 411 | السرطان وطرق علاجه  /store/apps/details?id=join.konbrand.Cancer2 |
| 412 | cancer du colon  /store/apps/details?id=com.andromo.dev667101.app687284 |
| 414 | Cancer Flashcards Plus  /store/apps/details?id=com.ablet.cancerflashcardsplus |
| 415 | Combat contre le cancer  /store/apps/details?id=com.traitementsAntiCancer.myandroapps |
| 416 | Physicians' Cancer Chemotherapy Drug Manual  /store/apps/details?id=com.atmoapps.pcch |
| 417 | Sepsis Clinical Guide  /store/apps/details?id=app.escavo.sepsis |
| 419 | Bladder Cancer  /store/apps/details?id=com.bedieman.bladderCancer |
| 420 | Cáncer de estómago: El cáncer gástrico  /store/apps/details?id=com.andromo.dev522931.app851685 |
| 421 | Anti Cancer Protocols  /store/apps/details?id=co.protocol.anticancer |
| 422 | Superfood Power  /store/apps/details?id=com.mobincube.superfood_power.sc_DW1TI7 |
| 423 | Los alimentos que combaten el cáncer  /store/apps/details?id=com.andromo.dev522931.app851668 |
| 424 | NCCN Guidelines for Smartphone  /store/apps/details?id=com.tipmedcom.nccn.guidelines.mobile |
| 425 | My Health Coach  /store/apps/details?id=com.hingerle.vorsorgemedizin |
| 426 | Cancer Flashcards Premium  /store/apps/details?id=com.mytabway.cancerflashcardspremium |
| 427 | ছোট রোগের ঘরোয়া চিকিৎসা  /store/apps/details?id=com.InnovativeBanglaApps.ChotoRogerGhorowaChikitsa |
| 428 | Cáncer de hueso  /store/apps/details?id=com.andromo.dev474745.app516863 |
| 429 | Fight Cancer Naturally  /store/apps/details?id=com.seawellsoft.fightcancerfree |
| 430 | Cara Alami Menyembuhkan Lipoma Secara Ampuh  /store/apps/details?id=com.ramuanherballipomapalingmanjurdanlengkap.obatherbal.kumpulanramuanherbal.mesotheliaomalawfirm |
| 431 | Cancer Prevention  /store/apps/details?id=com.bmooble.android.app.cancerprevention |
| 432 | NCCN Patient Guides for Cancer  /store/apps/details?id=com.mediaparts.nccn |
| 433 | bodyxq cancer  /store/apps/details?id=com.bodyxq.cancer |
| 434 | Dip Recipe  /store/apps/details?id=com.moboski.apps.android5a85955ca3eba |
| 435 | Endometrial Cancer  /store/apps/details?id=com.bedieman.endometrialCancer |
| 436 | Cara Alami Mengatasi Jantung Berdebar  /store/apps/details?id=com.ramuanherbaljantungberdebarlengkap.obatherbal.kumpulanramuanherbal.mesotheliaomalawfirm |
| 437 | Esteroides - Las fórmulas químicas de hormonas  /store/apps/details?id=com.asmolgam.steroids |
| 438 | Cancer du poumon  /store/apps/details?id=com.andromo.dev667101.app686642 |
| 440 | Jadi Butiyan \| जड़ी बुटिया - Gharelu Nuskhe, Ilaaj  /store/apps/details?id=com.app4daily.jadibutiya |
| 441 | Vitamins Need : विटामिन की जरुरत  /store/apps/details?id=com.srkphotography.vitamin |
| 442 | Cara Alami Mengobati Telinga Berjamur Dengan Mudah  /store/apps/details?id=com.caraalamimengobatitelingaberjamurlengkap.obatherbal.kumpulanramuanherbal.mesotheliaomalawfirm |
| 443 | Cancer testicular  /store/apps/details?id=com.Testicular.Cancer.Signs.Symptoms |
| 444 | Dadi Nani Maa Ka Khazana  /store/apps/details?id=com.app1daily.a4db404 |
| 445 | Cancer Guide Umeedein  /store/apps/details?id=info.kamaleshsenapati.umeedein |
| 446 | Cara Alami Mengobati Vertigo Atau Sakit Kepala  /store/apps/details?id=com.caraalamimengobativertigolengkap.obatherbal.kumpulanramuanherbal.mesotheliaomalawfirm |
| 447 | Super K vs Cancer  /store/apps/details?id=com.games.superKcontraCancer |
| 448 | Myriad MyRisk ™  /store/apps/details?id=com.infusemedical.myrisk |
| 449 | Consejos para la depresión, diabetes, corazón.  /store/apps/details?id=com.jbbd.fruitsandvegetables |
| 450 | Lucha contra el Cancer  /store/apps/details?id=com.andromo.dev616791.app872839 |
| 452 | Tips Menyembuhkan Telinga Gatal Bagian Dalam Ampuh  /store/apps/details?id=com.obatalamimengatasitelingagatallengkap.obatherbal.kumpulanramuanherbal.mesotheliaomalawfirm |
| 453 | Les rí©flexes anticancer au quotidien  /store/apps/details?id=com.zmoon.Anticancer |
| 455 | ZibdyHealth  /store/apps/details?id=com.zibdy.VPB.client |
| 456 | iURO Oncology  /store/apps/details?id=com.ACSimulation.iURO_Onco |
| 457 | Top Cancer Fighting Foods  /store/apps/details?id=com.andromo.dev685440.app754800 |
| 458 | Calculadora de edad perruna  /store/apps/details?id=com.amdroid.calculadora.edad.perruna |
| 459 | Glitter Cancer Constellation Theme & Wallpaper  /store/apps/details?id=com.apusapps.theme.i_constellation_cancer_b2ab4e1366 |
| 460 | Chay ke Nuksaan  /store/apps/details?id=com.mts.chaykenuksaan |
| 461 | Health benefits of Pumpkin Seeds  /store/apps/details?id=mmdigitalproducts.com.healthbenefitoPumpkinSeeds |
| 462 | सिर्फ महिलाओं के लिए(Onlygirl)  /store/apps/details?id=com.srkphotography.onlyforgirl |
| 463 | Tät III - Bäckenbottenträning  /store/apps/details?id=se.umu.its.tatman |
| 465 | কাঁচা ও পাকা আমের পুষ্টিগুণ ও উপকারিতা  /store/apps/details?id=com.itjogot.benefiteofmango |
| 467 | A to Z rogo ke upay(Diseases)  /store/apps/details?id=com.ltbphotoframes.atozrogokeupay |
| 468 | Cara Mengobati Telinga Bernanah Dengan Bahan Alami  /store/apps/details?id=com.obatalamimengatasitelingabernanahlengkap.obatherbal.kumpulanramuanherbal.mesotheliaomalawfirm |
| 469 | Manual of Laboratory & Diagnostic Tests Fischbach  /store/apps/details?id=com.medpresso.Lonestar.labtests |
| 470 | Homeopathy Treatment  /store/apps/details?id=com.kingofstoryapp.homeopathytreatment |
| 471 | Cancer Terms Dictionary  /store/apps/details?id=com.edictonary.CancerTerms.Dictionary |
| 473 | All In One Disease Dictionary  /store/apps/details?id=com.rogokeupay.diseasesdictionary |
| 474 | কাঁচা টমেটোর কিছু উপকারিতা  /store/apps/details?id=com.itjogot.useoftomato |
| 475 | التغذية السليمة نصائح و فوائد  /store/apps/details?id=com.societedeservice.ts |
| 476 | Untire: Beating cancer fatigue  /store/apps/details?id=com.tiredofcancer |
| 477 | Types of Cancer Treatment  /store/apps/details?id=com.andromo.dev712859.app826270 |
| 478 | Chay Pine Ke Nuksan- Side effects of drinking tea  /store/apps/details?id=com.fullfunapps.teasideeffects |
| 480 | Remedios prevención del Cáncer  /store/apps/details?id=com.andromo.dev708981.app888622 |
| 481 | 🌿 Remedios Caseros Para Todo  /store/apps/details?id=com.mobincube.remedios_caseros.sc_HHAJKC |
| 483 | Ramuan Herbal Mengobati Hidung Alergi Paling Ampuh  /store/apps/details?id=com.ramuanherbalmengobatihidungalergilengkap.obatherbal.kumpulanramuanherbal.mesotheliaomalawfirm |
| 484 | Enfermedad Renal Crónica  /store/apps/details?id=com.Treatmento.Kidneyyo |
| 485 | 國鼎生物科技  /store/apps/details?id=com.heimavista.magicsquare.joan1 |
| 486 | Cervical Cancer  /store/apps/details?id=com.bedieman.cervicalCancer |
| 487 | Varios jugos saludables  /store/apps/details?id=com.adhelliaapps.varioushealthyjuices |
| 488 | Cancer Cervix Fact  /store/apps/details?id=com.andromo.dev677250.app828817 |
| 489 | Ultrasound Guide  /store/apps/details?id=com.ultrasoundguide.healthylifestore |
| 490 | घरेलु नुस्खे सम्पूर्ण गाइड - Learn Home Remedies  /store/apps/details?id=com.sixtynintyapps.ghsxtynntykflex |
| 491 | Colon Cancer  /store/apps/details?id=com.coloncancer.goodapps |
| 492 | Урология и андрология  /store/apps/details?id=ru.volynka.urology.android |
| 493 | Anti cancer food! Cancer care! anti cancer diet!  /store/apps/details?id=com.rsnapp.cancer_protirodhok_khabar |
| 494 | Cancer-killing drug  /store/apps/details?id=com.andromo.dev712859.app847451 |
| 495 | Skin Cancer Symptoms  /store/apps/details?id=skin.cancer.symptoms |
| 497 | Lung Cancer Stage  /store/apps/details?id=com.cvoffice.lungcastage |
| 498 | Disease ( बीमारी ) bhagaye khan pan ( food ) se  /store/apps/details?id=com.bmari.khnpn |
| 499 | 腺ノート～前立腺がん患者さんの診察時コミュニケーション支援～  /store/apps/details?id=jp.sennote.app |
| 501 | Horóscopo Cáncer ♋ Diario Gratis  /store/apps/details?id=cancer.horoscope.daily.free |
| 502 | Cáncer ♋ Horóscopo del día 2019  /store/apps/details?id=com.adnfxmobile.discovery.cancer |
| 511 | Self-Care During Cancer  /store/apps/details?id=com.nearspace.selfcare |
| 514 | Kids' Guide to Cancer  /store/apps/details?id=com.twoandtwo.campquality |
| 518 | 8 SURAH UNTUK PENAWAR CANCER  /store/apps/details?id=com.andromo.dev405941.app383862 |
| 519 | Lucha contra el Cancer  /store/apps/details?id=com.andromo.dev616791.app872839 |
| 520 | Horóscopo Cáncer  /store/apps/details?id=com.IVR.horoscopocancer |
| 522 | SkinVision - Detect Skin Cancer  /store/apps/details?id=com.rubytribe.skinvision.ac |
| 524 | Cancer Horoscope - Cancer Daily Horoscope 2019  /store/apps/details?id=daily.horoscope.cancer |
| 525 | Testicular Cancer Self Exam  /store/apps/details?id=org.testicularcancersociety.android |
| 526 | Cancer  /store/apps/details?id=cancer.findyourfate.com |
| 529 | Intl. VTE & Cancer Guidelines  /store/apps/details?id=com.exolnet.itaccme |
| 532 | Cancer Conditions & Treatments  /store/apps/details?id=com.spaceO.cancerConditionsTreatments |
| 535 | Cancer Staging Tools  /store/apps/details?id=info.cancertools |
| 537 | FIGO Gyn Cancer Management  /store/apps/details?id=app.com.figostaging |
| 538 | Ways To Fight Off Cancer  /store/apps/details?id=com.koodalappz.cancercure |
| 539 | Prevent Cancer  /store/apps/details?id=com.silverfinger.preventcancer |
| 540 | TNM Cancer Staging Manual  /store/apps/details?id=com.medpresso.Lonestar.ajcc |
| 543 | PM Cancer Journey  /store/apps/details?id=ca.uhn.pmcpWebTeam.pmCancerJourney |
| 544 | Cancer Curing Foods  /store/apps/details?id=com.proven.cancercure.AOUJZCYXQQQEVGMK |
| 546 | CanCell Cancer  /store/apps/details?id=pl.gov.nfz.cancellcancer |
| 547 | The Ride to Conquer Cancer CAN  /store/apps/details?id=com.conquer.canada |
| 548 | DermIA - Análisis Cancer Piel manchas con Cámara  /store/apps/details?id=com.genialabs.medicaible |
| 552 | Molexplore - Melanoma & Skin Cancer App  /store/apps/details?id=com.borealos.medical.molexplore |
| 554 | Survive - an initiative by Win Over Cancer  /store/apps/details?id=woc.ngo |
| 557 | CanDi - Cancer Diet App  /store/apps/details?id=com.brok3n.candiapps.candiapps |
| 558 | Super Food to Fight for Cancer  /store/apps/details?id=com.cylonblastmobileapps.superfoodtofightcancer |
| 560 | Understanding Cancer  /store/apps/details?id=com.andromo.dev712859.app826317 |
| 562 | Cervical Cancer  /store/apps/details?id=com.cervical.cancer.app |
| 563 | European Journal of Cancer  /store/apps/details?id=com.elsevier.gmr.ejc |
| 567 | Cancer Horoscope  /store/apps/details?id=com.bhaee.cancer |
| 568 | cáncer lwp  /store/apps/details?id=ttr29cancer.lwp |
| 569 | ক্যান্সার \| cancer  /store/apps/details?id=com.canser.problem.solution.tips |
| 570 | Cancer Dojo  /store/apps/details?id=org.cancerdojo |
| 574 | Bible Verses For Cancer Patient - Strength Verses  /store/apps/details?id=com.bibleversesforcancerpatient |
| 581 | Esophageal Cancer  /store/apps/details?id=com.bedieman.esophagealCancer |
| 584 | Early Symptoms of Cancer  /store/apps/details?id=xyz.cursedapps.cancer |
| 589 | CANCER: THE GAME  /store/apps/details?id=com.PoppletonGames.Cancer |
| 591 | Basil OncoCare,Cancer Hospital  /store/apps/details?id=com.oozeetech.basil |
| 594 | Cáncer de tiroides  /store/apps/details?id=com.andromo.dev474745.app540393 |
| 595 | CancerAid  /store/apps/details?id=au.com.canceraid |
| 597 | Cancer iChart  /store/apps/details?id=com.liverpooluni.ichartoncology |
| 599 | ক্যান্সার Cancer ~ রোগ ও চিকিৎসা  /store/apps/details?id=com.ayaatapps.cancer_info |
| 602 | Cáncer Zodiaco â™‹ Teclado Tema Cangrejo  /store/apps/details?id=com.constellation.keyboard.theme.cancer |
| 603 | Scottish Cancer Referral Guidelines  /store/apps/details?id=com.scet.cancercareguidelines |
| 607 | Cancer Genetics  /store/apps/details?id=com.ubqo.cancergenetics |
| 609 | Mapa Tus Lunares - MySkinPal  /store/apps/details?id=com.masseranolabs.myskinpal |
| 612 | Lung Cancer Staging Table  /store/apps/details?id=air.stagingandroid |
| 613 | স্তন ক্যান্সার সচেতনতা Breast Cancer  /store/apps/details?id=com.ertapps.breast_cancer_new |
| 614 | Know Cancer  /store/apps/details?id=com.knowcancer.hnnh.herokuapp |
| 615 | MVR Cancer Centre  /store/apps/details?id=org.mvrccri.app |
| 616 | Miiskin - Cáncer de piel  /store/apps/details?id=com.miiskin.android |
| 619 | লিভার কান্সার চিকিত্‍সা ~ Liver cancer Treatment  /store/apps/details?id=com.Medical.Disease.Treatment.Liver_cancer |
| 621 | Les aliments qui causent le cancer  /store/apps/details?id=com.andromo.dev667101.app678336 |
| 624 | Cancer Daily Horoscope for Today with Love & Money  /store/apps/details?id=cancer.daily.horoscope |
| 628 | GRYT Health Cancer Community  /store/apps/details?id=com.grytapp |
| 629 | Soursop Health Benefit: Anti Cancer Super Fruit  /store/apps/details?id=cancer.anticancer.soursop.health.medicine |
| 630 | Diccionario de Cáncer  /store/apps/details?id=com.appseducativos.diccionariodecancer |
| 633 | LungScreen  /store/apps/details?id=hu.markcon.lungtumourfilter |
| 634 | Cancer Emoji Keyboard Theme  /store/apps/details?id=com.kkkeyboard.emoji.keyboard.theme.Cancer |
| 642 | Beat Cancer  /store/apps/details?id=com.Polyspice.BeatCancer |
| 643 | Cervical Cancer  /store/apps/details?id=com.Cervical.cancer |
| 644 | Endometrial Cancer  /store/apps/details?id=com.endometrial.cancer.app |
| 645 | Anti Cancer Foods  /store/apps/details?id=com.anti.cancer.foods |
| 648 | Cancer Genomics & Proteomics Journal  /store/apps/details?id=com.slidescenter.journalapp.cgp |
| 649 | 10 Ways To Fight Cancer  /store/apps/details?id=com.bookfreeapps.fightcancer |
| 651 | How To Cure Liver Cancer  /store/apps/details?id=com.howto.howtocurelivercancer |
| 654 | Ovarian Cancer Awareness  /store/apps/details?id=com.magna.ovariancancer |
| 655 | ক্যান্সার থেকে মুক্তির উপায়-Cancer  /store/apps/details?id=com.arrstudio.cancer_theke_muktir_upay |
| 657 | Cura el cáncer de Nadia  /store/apps/details?id=com.protectfive.korner.xplorehealth.curenadia |
| 659 | Melanoma Detection  /store/apps/details?id=com.bakten.skincancer |
| 660 | leucémie  /store/apps/details?id=com.andromo.dev667101.app681152 |
| 662 | Cancer Plantas  /store/apps/details?id=cancer.salud.enfermedad.AOVAGCQHQILAVZCZ |
| 663 | Fight Cancer Boldly: Game (Free)  /store/apps/details?id=com.gforgoodness.fightcancerboldlyfree |
| 665 | Lung Cancer Staging 8th Edition  /store/apps/details?id=com.lungcancerstaging |
| 666 | MD Anderson Mobile  /store/apps/details?id=com.mymda |
| 668 | The Ride to Conquer Cancer BC  /store/apps/details?id=com.conquer.cfridecav |
| 669 | How To Cure Cancer  /store/apps/details?id=com.howto.howtocurecancer |
| 670 | IASLC Staging Handbook  /store/apps/details?id=com.iaslc.staging |
| 672 | ESGO Gynae Cancers Algorithms  /store/apps/details?id=com.esgomobileguides.org |
| 673 | Recognize Signs of Oral Cancer  /store/apps/details?id=com.recognize.signs.of.oral.cancer.achtech007 |
| 675 | Alimentos contra el cáncer - Tips & FAQ  /store/apps/details?id=com.proyectoultra8 |
| 676 | Alimentation Anti Cancer  /store/apps/details?id=com.alimentation.anti.cancr |
| 677 | Cancer ♋ Daily Horoscope 2019  /store/apps/details?id=com.vitalapps4.daily.free.horoscope.cancer |
| 679 | Top 10 Cancer Fighting Foods  /store/apps/details?id=com.t_999appsdeveloper.Top_10_Cancer_Fighting_Foods |
| 680 | El Cáncer  /store/apps/details?id=com.el.cancer |
| 681 | Horóscopo Cáncer  /store/apps/details?id=com.LogOvoide.Horoscope_Cancer |
| 682 | CA:Cancer Journ for Clinicians  /store/apps/details?id=com.wiley.mobile.jas.caac |
| 685 | Cancer Congress 2019  /store/apps/details?id=com.tentimes.cancercongressbel |
| 686 | Zodiac Cáncer Go Keyboard  /store/apps/details?id=com.jb.gokeyboard.theme.mmthemes.cancer |
| 689 | Shivmani Cancer Hospital  /store/apps/details?id=com.nirvanza.drshaileshspatel |
| 690 | blood cancer  /store/apps/details?id=com.andromo.dev670419.app693277 |
| 691 | 전립선암 계산기  /store/apps/details?id=org.snubh.prostate_calc |
| 692 | Cancer Horoscope Home - Daily Zodiac Astrology  /store/apps/details?id=com.home.horoscope.cancer.theme |
| 693 | NCCN Guidelines®  /store/apps/details?id=com.tipmedcom.nccn.guidelines |
| 694 | Las plantas de hierbas previenen el cáncer  /store/apps/details?id=com.JujuDroid.ReferenceHerbalCancerPrevesion |
| 695 | Colon Cancer Coalition  /store/apps/details?id=com.charitydynamics.ccc |
| 696 | Target Ovarian Cancer Symptoms Diary  /store/apps/details?id=uk.org.targetovariancancer.symptomdiary |
| 697 | Recognize Stomach Cancer  /store/apps/details?id=mediaclinic.recognizestomachcancer |
| 698 | Ovarian Cancer Symptoms Diary  /store/apps/details?id=com.ionicframework.ocasymptomdiaryapp437535 |
| 699 | Cancer Treatment Tips  /store/apps/details?id=com.Cancertreatmenttips |
| 700 | Le cancer du temps  /store/apps/details?id=ca.onf.cancerdutemps |
| 701 | Guí­a pacientes Cáncer  /store/apps/details?id=com.clinicadelcountry.oncologia |
| 702 | Recognize Symptoms of Throat Cancer  /store/apps/details?id=com.recognize.symptoms.of.throat.cancer.achtech007 |
| 703 | Cancer Ka Ilaj Offline  /store/apps/details?id=com.IslamGhar.CancerKaIlaj |
| 704 | CANCER AND NATURAL TREATMENTS 2019  /store/apps/details?id=com.cancerandtreatment.app |
| 705 | Cancer Biology and Immunology  /store/apps/details?id=com.tentimes.cancerbiologyandimmunology |
| 706 | Cervical cancer Information  /store/apps/details?id=com.dcitltd.dcitltd.cervicalcancerinfo |
| 707 | All Skin Diseases and Treatment- A to Z  /store/apps/details?id=com.patrikat.skindiseases |
| 708 | NCI@NIH Fellows & Young Investigators  /store/apps/details?id=com.bluepanestudio.nci_fyi_2011 |
| 710 | Lung Cancer Information And Support  /store/apps/details?id=com.thereyv.lungcancer |
| 713 | Daily Horoscope Plus ® - Zodiac Sign and Astrology  /store/apps/details?id=com.daily.horoscope.plus |
| 714 | Phalo Se Cancer Ka Ilaj  /store/apps/details?id=com.appnextdoor.Cancer |
| 715 | Cancer  /store/apps/details?id=com.cancerdata.cancerdrugs |
| 716 | 365CPS  /store/apps/details?id=us.originally.cancer365 |
| 720 | 72 Recetas Anticancer/mis recetas anticancer  /store/apps/details?id=com.appstudio7.recetasanticancer |
| 721 | Cancer Awareness Network  /store/apps/details?id=com.app_canceranc.layout |
| 722 | Cancer 3D  /store/apps/details?id=com.sunrisevr.Cancer |
| 723 | Cancer Science App for Android  /store/apps/details?id=com.wiley.jas.cas |
| 724 | Esophageal Cancer  /store/apps/details?id=com.esophageal.cancer.app |
| 725 | Qatar Cancer Society  /store/apps/details?id=com.applab.QCS |
| 726 | Swastava cancer care - society  /store/apps/details?id=com.wingherrytechnologies.swastavacancercareupdate |
| 727 | Reachout: My Support Network  /store/apps/details?id=com.reachout |
| 728 | Cancer Theme - Wallpapers and Icons  /store/apps/details?id=com.wxyz.theme.cancer |
| 729 | Cáncer fondos pantalla en vivo  /store/apps/details?id=com.awesomelivewallpapers.cancerlivewallpapers |
| 731 | Pancreatic Cancer Action - symptom tracker  /store/apps/details?id=com.healthbit.pca |
| 732 | ASCO Guidelines  /store/apps/details?id=org.asco.guidelines |
| 733 | My Care Plan (cancer survivor)  /store/apps/details?id=com.nearspace.mycareplan |
| 735 | St Peregrine Patron of Cancer Patients Novena  /store/apps/details?id=com.newjapps.stperegrinenovena |
| 739 | Colon Cancer Info  /store/apps/details?id=com.programmingisfun.coloncancer |
| 740 | Acute Oncology Support  /store/apps/details?id=com.velindrenhstrust.aoswales |
| 742 | ক্যান্সার এর লক্ষণ ও করণীয়  /store/apps/details?id=com.itjogot.cancersymptoms |
| 743 | Tratamiento del cáncer con fruta de Huaya  /store/apps/details?id=com.itatvadev.tratamientodelcancerconfrutadehuaya |
| 745 | Cancer Horoscope Free Keyboard Theme  /store/apps/details?id=cancer.horoscope.free.keyboard.theme |
| 746 | Cancer jadu Aur Dosri Bemarion Ka Asan Ilaj  /store/apps/details?id=sadafent.CancerjaduAurDosriBemarionKaAsanIlaj |
| 747 | How to Detect Cancer Early  /store/apps/details?id=com.andromo.dev717872.app811937 |
| 748 | Frases para Amigos con Cáncer  /store/apps/details?id=com.cerezapps.frasesparaamigosconcancer |
| 753 | Colon Cancer  /store/apps/details?id=com.coloncancer.myandroapps |
| 754 | Colon Cancer Screening/Education App  /store/apps/details?id=com.notexample.emmet.coloncancerscreening |
| 756 | CaPtyVa - Pesquisa y Vigilancia Cáncer Colorrectal  /store/apps/details?id=biz.app4mobile.app_f9a3be4f14124c54a4746161767c6135.app |
| 758 | ALIANZA COLON  /store/apps/details?id=com.render.alianzacolonAndroid |
| 759 | Cancer Colon  /store/apps/details?id=com.CancerColon.smartapps |
| 760 | Colon Cancer Awareness  /store/apps/details?id=com.coloncancer.smartapps |
| 761 | CRC onkowissen  /store/apps/details?id=de.onkowissen.crc |
| 762 | Alternative Therapy For Colon Cancer  /store/apps/details?id=com.seawellsoft.coloncancerexp |
| 763 | ScreenTool: Colorectal Cancer  /store/apps/details?id=com.mobincube.screentool_colorectal_cancer.sc_3KWPXQ |
| 764 | Colo-Rectal Cancer Screening  /store/apps/details?id=com.mobincube.resident_guide_for_colo_rectal_cancer_screening.sc_HDJGDW |
| 765 | Sổ Tay Phòng Bệnh  /store/apps/details?id=vn.phongbenh.ungthu |
| 766 | Colon Cancer  /store/apps/details?id=com.healthyvisions.coloncancer |
| 768 | Limpiar el Colon  /store/apps/details?id=com.jugos.para.limpiar.el.colon.naturales.frutas.verduras |
| 773 | Colon Cancer  /store/apps/details?id=com.bmooble.android.app.colonCancer |
| 774 | 대장암 by 세컨드 닥터  /store/apps/details?id=com.mediplussolution.android.csmsrenewal.colorectalcancer |
| 775 | Share It Colorectal Cancer  /store/apps/details?id=com.deepak.doctors |
| 776 | PrevColon  /store/apps/details?id=com.seisunos.prevcolon |
| 777 | Cancer de Colón  /store/apps/details?id=com.mmi.colorectalcancer |
| 782 | 《醫藥人》第168期  /store/apps/details?id=com.medcom.yiyaoren168 |
| 783 | Darmkrebs CheckApp Shared Decision Making  /store/apps/details?id=com.fabware.darmcheck |
| 784 | Dr. Saldaña  /store/apps/details?id=com.mobincube.dr_saldana.sc_HJTYU4 |
| 787 | Navina Smart  /store/apps/details?id=com.dentsply.smart |
| 788 | ক্যান্সার সচেতনতা~Cancer Awareness  /store/apps/details?id=com.noorapps.cancer_awareness |
| 789 | Cáncer de esófago  /store/apps/details?id=com.andromo.dev474745.app553517 |
| 791 | علاج بومزوي (القولون العصبي)  /store/apps/details?id=com.ilaj_kolon_asabi_bomzwi.amrad_al_masran_albatn_colon |
| 792 | Рак кишечника  /store/apps/details?id=com.appglobus.app.android591aa861624e8as |
| 793 | Bowel Cancer info WMUH  /store/apps/details?id=com.appmakr.colorectalsurgerywestmiddlesexuniversityhospital |
| 794 | ColorApp  /store/apps/details?id=com.bigapps.colorapp1 |
| 799 | Limpieza Natural del Colon  /store/apps/details?id=com.JuBat.naturalcoloncleanse |
| 800 | COLONCOP  /store/apps/details?id=com.imaidea.norgine.coloncop |
| 801 | Poop Tracker - Toilet Log  /store/apps/details?id=com.appstronautstudios.pooplog |
| 802 | कैंसर Guide  /store/apps/details?id=com.cancerguide.pala |
| 803 | Disease and Prevention  /store/apps/details?id=com.redgrenache.diseaseandprevention |
| 805 | รู้ทันป้องกันมะเร็งลำไส้ใหญ่  /store/apps/details?id=colon.cancer.peventionapp |
| 806 | CRCbox  /store/apps/details?id=com.connectmedica.crcbox |
| 810 | David Omenukor Foundation  /store/apps/details?id=com.tigerappcreator.cms.android5881da75061a7 |
| 812 | Gastroenterology Basics  /store/apps/details?id=com.andromo.dev738202.app854430 |
| 821 | Casos Clínicos Regorafenib  /store/apps/details?id=com.ccregorafenib.app |
| 822 | Doç. Dr. Gökhan Çipe  /store/apps/details?id=mobturkiye.gokhancipe |
| 823 | علاج القولون العصبي مجرب  /store/apps/details?id=com.ailajudag.kaloinh |
| 825 | EFR  /store/apps/details?id=com.e_materials.efr |
| 827 | Oncoscreen  /store/apps/details?id=kz.ergaliev.zhaslan.searchistkaz |
| 828 | Easy RAS  /store/apps/details?id=com.aumenta.merck |
| 832 | cancer prevention  /store/apps/details?id=com.njo.cancer.foods.info |
| 837 | Santé  /store/apps/details?id=com.andromo.dev518100.app634453 |
| 838 | Recetas de jugos medicinales sencillos de preparar  /store/apps/details?id=com.blancoynegrostudios.jugosmedicinales01 |
| 839 | السرطان وطرق علاجه  /store/apps/details?id=join.konbrand.Cancer2 |
| 843 | ISUCRS 2016  /store/apps/details?id=com.gephels.isucrs |
| 845 | APP REBECCA  /store/apps/details?id=com.bayer.ph.apprebecca |
| 846 | Detox Colon Cleanse  /store/apps/details?id=com.tototomato.detoxcoloncleanse |
| 847 | Constipation Info  /store/apps/details?id=com.programmingisfun.constipation |
| 849 | Constipation Diagnosis Doctor  /store/apps/details?id=com.easydiagnosis.constipation |
| 850 | GASTRO KV 2018  /store/apps/details?id=com.tri.gcon.gastro2018 |
| 852 | 黃正宏家庭親子診所  /store/apps/details?id=tw.com.hostingservice24.dr_growth |
| 855 | علاج سرطان الثدي  /store/apps/details?id=tady.saratan.ilaj.com |
| 856 | ピロリ菌除菌と胃がん予防についてクイズで学ぼう  /store/apps/details?id=net.jp.apps.koikonishi.piroriqu |
| 857 | Constipation Disease  /store/apps/details?id=com.bedieman.constipationDisease |
| 858 | علاج أمراض القولون  /store/apps/details?id=apps.konbrand.QaolonNewStyle |
| 860 | Beans For Health  /store/apps/details?id=com.extwebtech.beansforhealth |
| 861 | জেনে নিন ভুট্টার দারুণ স্বাস্থ্য উপকারিতা  /store/apps/details?id=com.itjogot.cornbenefits |
| 862 | Health benefits of Cherry  /store/apps/details?id=mmdigitalproducts.com.healthbenefitCherry |
| 863 | Gastrointestinal USMLE S2CK QA  /store/apps/details?id=com.topoflearning.free.medical.apps.gastrointestinal.usmle.ck.science.flashcards |
| 864 | طبيب العائلة ـ علاج السرطان  /store/apps/details?id=km.app.FamilyDoctorCancer |
| 865 | BELLY FAT BURN EXERCISES FOR MEN  /store/apps/details?id=bellyfat.exercises.men |
| 866 | Iron-deficiency Anemia Info  /store/apps/details?id=com.programmingisfun.irondefanemia |
| 867 | Gastrointestinal USMLE Stp2 CK  /store/apps/details?id=com.topoflearning.best.medical.apps.gastrointestinal.usmle.ck.science.flashcards |
| 868 | কোষ্ঠকাঠিন্য রোগের চিকিৎসা ~Constipation Treatment  /store/apps/details?id=com.Medical.constipation.Disease.Treatment |
| 869 | All Stomach Diseases and Treatment  /store/apps/details?id=com.patrikat.stomachdiseasesandtreatment |
| 871 | Tratamientos y Enfermedades  /store/apps/details?id=com.Treatmento.diseasesALL |
| 872 | Encourage Health  /store/apps/details?id=com.axialexchange.invitation_app |
| 873 | Crohns Disease  /store/apps/details?id=com.bedieman.crohnsDisease |
| 874 | Colorectal Aid Operation guide  /store/apps/details?id=appinventor.ai_surgicalreg.Colorectal_Aid |
| 876 | العين (امراض العيون ،اعراض القولون ،كيفية تصحيح)  /store/apps/details?id=com.kolma.osblain |
| 877 | Swasti Gastroenterology Surgery Center  /store/apps/details?id=com.swasti.sweta.swasti |
| 878 | Simple Ways to Prevent Cancer  /store/apps/details?id=com.andromo.dev687069.app853574 |
| 879 | Miiskin - Cáncer de piel  /store/apps/details?id=com.miiskin.android |
| 881 | Health benefits of Honeyberry  /store/apps/details?id=mmdigitalproducts.com.healthbenefitofHoneyberry |
| 882 | Health Benefits Of Coffee  /store/apps/details?id=com.tadapps.health.benefits.of.coffee |
| 883 | نصائح ومعلومات مرض السرطان 2018  /store/apps/details?id=com.canser.alkasergase |
| 885 | Healthline Official App by WHO  /store/apps/details?id=com.t_999appsdeveloper.Healthline_Official_App_by_WHO |
| 886 | علاج القولون العصبي  /store/apps/details?id=com.torok.mojaraba.licifae.lkolon.l3assabi |
| 887 | Clini-Data™  /store/apps/details?id=com.scymed.android.ehnp |
| 888 | Recetas con 🍛 arroz 🌾insuperables yGratis  /store/apps/details?id=com.andromo.dev742675.app921391 |
| 889 | علاج منزلية لحرقة المعدة والحموضة  /store/apps/details?id=com.kerhatmarida.homouda |
| 890 | IOSI Linee Guida  /store/apps/details?id=ch.eoc.lineeguidaiosi |
| 893 | Food Benefits 2019  /store/apps/details?id=com.FoodBenefits12.FoodBenefits12 |
| 899 | Proven Benefits of Olive Oil  /store/apps/details?id=com.andromo.dev717872.app766748 |
| 907 | Cancer Types In Women  /store/apps/details?id=com.healthisveryimportant.cancertypesinwomen |
| 909 | Cocina de champií±ones  /store/apps/details?id=com.CuisineOfMushrooms.fahmidromo |
| 912 | Hemorrhoids Treatment - How To Get Rid Of Piles  /store/apps/details?id=hemorrhoids.treatment.piles.cure |
| 913 | علاج مفيد لأمراض الجهاز التنفسي  /store/apps/details?id=com.jihaztanfosi.dayke |
| 915 | Common Medical Imagings  /store/apps/details?id=com.andromo.dev677432.app888090 |
| 916 | No a las enfermedades, prevengamos el cancer  /store/apps/details?id=com.medicine.checkeate |
| 919 | Medgic - Analiza y detecta enfermedades de la piel  /store/apps/details?id=co.medgic.medgic |
| 922 | Лечащий Врач. Научный журнал  /store/apps/details?id=net.magtoapp.viewer.attendingdoc |
| 923 | Peso Saludable  /store/apps/details?id=com.peqconsultores.rimac |
| 926 | التخلص من غازات البطن  /store/apps/details?id=comp.Kayfyat_3ilaj_wa_izalat_Ghazat_Elbatn |
| 927 | Whats Web  /store/apps/details?id=com.whatsweb.app |
| 931 | Licuados y Jugos Saludables  /store/apps/details?id=com.andresapps.jugosmedicinales |
| 936 | Remedios caseros medicina natural alternativa  /store/apps/details?id=com.appsamimanera.remedioscaserosmedicinanatural |
| 937 | Diccionario de tratamiento de enfermedades  /store/apps/details?id=com.in_so.medical.disease.dictionary.free.offline |
| 938 | Effects Of Alcohol  /store/apps/details?id=com.sportzoon.effectsofalcohol |
| 939 | Toilet diary  /store/apps/details?id=com.stoelgangapp.meter.engels |
| 941 | The Secret Life of Aspirin  /store/apps/details?id=com.mbspringer.aspirin |
| 944 | Intussusception Disease  /store/apps/details?id=com.bedieman.intussusceptionDisease |
| 945 | Bolsa Empleo SAS 2.0  /store/apps/details?id=es.jcajrc.ibolsatrabajosas20 |
| 947 | All Stomach Diseases and Treat  /store/apps/details?id=com.StomachDiseasesTreat.smartapp |
| 949 | Síntomas y Enfermedades  /store/apps/details?id=sintomas.enfermedades |
| 950 | Constipation: Causes, Diagnosis, and Treatment  /store/apps/details?id=com.healthinfo.constipation.cause.prevention.management.treatment.diagnosis |
| 951 | 117 आयुर्वेदिक घरेलु इलाज  /store/apps/details?id=com.ayurvedicghareluilaaz117.pala |
| 953 | Estreñimiento 🆘 remedios caseros  /store/apps/details?id=com.TecnovApp.Estrenimiento |
| 954 | Gastrointestinal Diseases  /store/apps/details?id=com.medhand.EGDMA |
| 955 | কাঁচা ও পাকা আমের পুষ্টিগুণ ও উপকারিতা  /store/apps/details?id=com.itjogot.benefiteofmango |
| 956 | Iron Deficiency Anemia  /store/apps/details?id=com.bedieman.ironDeficiencyanemia |
| 957 | Dr. Ishan Shah  /store/apps/details?id=com.nirvanza.drishanshah |
| 959 | Whats Web Scan  /store/apps/details?id=com.hemanteditor.whatsweb |
| 960 | ক্যান্সার প্রতিরোধের খাবারসমূহ  /store/apps/details?id=com.itjogot.cancerpreventionmeal |
| 962 | Vegetables wallpaper  /store/apps/details?id=com.singhvi.Vegetableswallpaper |
| 964 | Maneras de combatir la obesidad.  /store/apps/details?id=com.Treatmento.Obesityy |
| 965 | علاج أمراض الجهاز التنفسي‎ بدون نت  /store/apps/details?id=join.jihaz.tanafossi |
| 966 | Fight CRC Late-Stage MSS CRC Trial Finder  /store/apps/details?id=org.fightcrc.trialfinder |
| 967 | Physical Examination & History Taking 8e  /store/apps/details?id=com.medhand.wkpeht8x1 |
| 968 | 健康好站-台北防癌一指通  /store/apps/details?id=org.ourcitylove.friendlycancer |
| 969 | Jugoterapia para la salud  /store/apps/details?id=jugoterapia.salud |
| 977 | Amgen Detect  /store/apps/details?id=com.technohat.ras |
| 985 | The Neurosurgical Atlas  /store/apps/details?id=com.neurosurgicalatlas.neurosurgicalatlasapp |
| 988 | Cadastro Cirurgia Colorretal  /store/apps/details?id=com.zumerata.formccc |
| 993 | atlasGO  /store/apps/details?id=com.atlasclient |
| 994 | Zero Calories seguidor de ayuno para perder peso  /store/apps/details?id=com.zelo_fasting.zelo |
| 995 | Water Reminder - Daily Water Tracker  /store/apps/details?id=com.water.daily.reminder.tracker |
| 997 | CURRENT Medical Diagnosis and Treatment 2019  /store/apps/details?id=com.medpresso.Lonestar.cmdt |
| 999 | Diseases Treatments Dictionary  /store/apps/details?id=com.diseases.treatment_dictionary |
| 1004 | IX Curso Avances Cáncer de Pulmón  /store/apps/details?id=com.tufabricadeventos.IXCursoAvancesCancerDePulmon |
| 1005 | Tratamientos Del Cáncer De Pulmón - FAQ  /store/apps/details?id=com.proyectoultra3 |
| 1009 | Lung Cancer  /store/apps/details?id=com.droidmedic.lungcancer |
| 1011 | Lung Cancer  /store/apps/details?id=com.epicsol.lungcancer |
| 1012 | Clean your lungs  /store/apps/details?id=com.dhadbadati.apps.clean_your_lung |
| 1014 | MeVis Lung-RADS  /store/apps/details?id=de.mevis.lungRADS |
| 1018 | FAQs in Lung Cancer  /store/apps/details?id=com.focusmedica.faq.lungcancer |
| 1023 | Quick Stage -Lung  /store/apps/details?id=com.tnm.surgery1appli |
| 1024 | All Respiratory Disease and Treatment  /store/apps/details?id=com.patrikat.respiratorydiseaseandtreatment |
| 1025 | 12th Congress on Lung Cancer  /store/apps/details?id=com.eventwo.lungc2017 |
| 1026 | TNM Lung Staging  /store/apps/details?id=gr.lectusadv.tnmstaging |
| 1027 | Mesothelioma Cancer  /store/apps/details?id=com.mesotheliomacancersymptoms.myandroapps |
| 1028 | Mesotelioma quimioterapia estadificación tumores  /store/apps/details?id=org.wikipedia.en.wiki.mesothelioma.cancer.lung.asbestos.app |
| 1034 | IASLC WCLC 2018  /store/apps/details?id=com.webges.wclc |
| 1035 | Humo  /store/apps/details?id=com.free.smoke.live.wallpapers.pretty.and.cute.wallpapers |
| 1038 | Lung Cancer  /store/apps/details?id=com.bmooble.android.app.lungCancer |
| 1039 | ক্যান্সার প্রতিরোধের খাবারসমূহ  /store/apps/details?id=com.itjogot.cancerpreventionmeal |
| 1040 | 《醫藥人》第187期  /store/apps/details?id=com.medcom.yiyaoren187 |
| 1043 | Fumar cigarrillo electrónico (PRANK)  /store/apps/details?id=us.virtualsmoking.ecigarette |
| 1045 | MRCP  /store/apps/details?id=fr.patientsenreseau.mrcp |
| 1047 | Respiratory diseases & respiratory therapy  /store/apps/details?id=com.respiratory.app |
| 1050 | How to Fight Lung Inflammation  /store/apps/details?id=com.andromo.dev717872.app809542 |
| 1054 | IASLC World Conference  /store/apps/details?id=com.coreapps.android.followme.iaslc_conferences |
| 1057 | MRCG  /store/apps/details?id=fr.patientsenreseau.mrcg |
| 1060 | 第58回 日本肺癌学会学術集会 My Schedule  /store/apps/details?id=jp.co.miceone.myschedule.jlcs2017 |
| 1061 | X-Ray Interpretation Guide  /store/apps/details?id=com.andromo.dev706301.app757834 |
| 1063 | علاج سرطان الثدي  /store/apps/details?id=tady.saratan.ilaj.com |
| 1065 | Mesothelioma Law  /store/apps/details?id=livecricketapps.mesotheliomalaw.cancerdisease |
| 1068 | ধূমপান থেকে বাঁচতে আদা  /store/apps/details?id=com.boishakhiapps.DhumpanThekeBachte |
| 1069 | RadioModels  /store/apps/details?id=ml.radiotherapy.radiomodels |
| 1070 | Народная медицина 2019  /store/apps/details?id=vasili.narodnaya.medicinanewver |
| 1072 | Sudoku Agentes Cancerí­genos  /store/apps/details?id=com.mutuauniversal.sudokucancerigenos |
| 1074 | Народные рецепты здоровья и красоты  /store/apps/details?id=ru.ymka.medic |
| 1077 | Ausculta Cardio-Pulmonar  /store/apps/details?id=br.com.noiawilsonferraz.auscultar |
| 1078 | লিভার কান্সার চিকিত্‍সা ~ Liver cancer Treatment  /store/apps/details?id=com.Medical.Disease.Treatment.Liver_cancer |
| 1079 | Beneficios de dejar de fumar.  /store/apps/details?id=beneficiosdejarde.fumar.benefitsquitsmoking |
| 1084 | ফুসফুস ক্যানসারের ৮টি লক্ষণ  /store/apps/details?id=com.boishakhiapps.FusfusKencharerlokon |
| 1088 | कैंसर Guide  /store/apps/details?id=com.cancerguide.pala |
| 1089 | Clonify.online  /store/apps/details?id=online.clonify.paciente |
| 1090 | 대한폐암학회 학술대회 공식 어플리케이션  /store/apps/details?id=com.guidebook.apps.Lungca.android |
| 1091 | Surgical Anatomy of the Lung  /store/apps/details?id=com.emory.lungapp |
| 1094 | Respiratory (Pulmonology) Calculators  /store/apps/details?id=com.respiratory.calculators |
| 1095 | Pulmonary Nodules - Fleishner 2017 Calculator  /store/apps/details?id=com.project_tl.pulmnod |
| 1096 | Treatments for Diseases  /store/apps/details?id=com.diseases.treatments |
| 1097 | ক্যান্সার সচেতনতা~Cancer Awareness  /store/apps/details?id=com.noorapps.cancer_awareness |
| 1100 | IASLC Atlas ALK & ROS1 Testing  /store/apps/details?id=com.b24.atlas |
| 1102 | Народная медицина - Про версия  /store/apps/details?id=medapps.info.medicinapro |
| 1109 | السرطان وطرق علاجه  /store/apps/details?id=join.konbrand.Cancer2 |
| 1110 | طبيب العائلة ـ علاج السرطان  /store/apps/details?id=km.app.FamilyDoctorCancer |
| 1113 | Домашний Доктор (без рекламы)  /store/apps/details?id=ru.ymka.medicpro |
| 1115 | Sistema respiratorio: enfermedades y tratamientos  /store/apps/details?id=com.Treatmento.Resporyy |
| 1116 | Asma  /store/apps/details?id=com.healthcareit.Asthma.Attack |
| 1118 | Asbestos  /store/apps/details?id=asbestos.com.blogspot.codedeveloped.asbestos |
| 1121 | OncoVision  /store/apps/details?id=appinventor.ai_viraj28m.CancerApp |
| 1124 | Cigarrillos Cigarettoid GRATIS  /store/apps/details?id=com.mmtlabs.gcigarettoid |
| 1126 | Beneficios De Dejar de Fumar  /store/apps/details?id=stopsmoking.dejardefumar.deixardefumar |
| 1128 | Síntomas  /store/apps/details?id=andy.symptomes |
| 1129 | recetas de jugo de salud  /store/apps/details?id=com.rellyoapps.healthjuicerecipes |
| 1132 | Medical Dictionary offline terms definitions 2019  /store/apps/details?id=com.brainappsville.medicaldictionary |
| 1134 | Greenplanet - Salva el planeta, planta un árbol!  /store/apps/details?id=com.greenplanet.plantatree |
| 1137 | Dejar de fumar - Dejar de fumar  /store/apps/details?id=com.stopsmoking.quitsmoking.nonsmoker |
| 1139 | Cráneo que fuma  /store/apps/details?id=com.smoking.skull.live.wallpaper.pretty.and.cute.wallpapers |
| 1142 | ピロリ菌除菌と胃がん予防についてクイズで学ぼう  /store/apps/details?id=net.jp.apps.koikonishi.piroriqu |
| 1144 | Mesothelioma Asbestos Cancer  /store/apps/details?id=com.einslabs.mesothelioma.lungcancer |
| 1147 | Calculate by QxMD  /store/apps/details?id=com.qxmd.calculate |
| 1149 | Repara tus rií±ones  /store/apps/details?id=com.imappz.kidneyremedies |
| 1150 | MilMed  /store/apps/details?id=com.ejemplo.milmed |
| 1151 | نصائح ومعلومات مرض السرطان 2018  /store/apps/details?id=com.canser.alkasergase |
| 1152 | Diseí±o de piso de cerámica  /store/apps/details?id=com.ceramicfloordesign.farah |
| 1154 | Más de 1000 ideas de diseí±o de talla de  /store/apps/details?id=com.chronosstudio.a1000StoneCarvingDesignIdeas |
| 1157 | Mesothelioma Cancer  /store/apps/details?id=com.mesotheliomacancersymptoms.goodapp |
| 1158 | English Medical Abbreviations Offline Dictionary  /store/apps/details?id=com.Smartcentre.medical_abbreviations |
| 1159 | DNA Analyzer  /store/apps/details?id=com.dnaanalyzer |
| 1160 | HOW TO STOP SMOKING  /store/apps/details?id=howto.stop.smoking.quit |
| 1161 | Cómo dejar de fumar  /store/apps/details?id=com.leontechnologies.HowToQuitSmoking |
| 1164 | Mouth Cancer  /store/apps/details?id=com.bedieman.mouthCancer |
| 1165 | ILCSC 2019  /store/apps/details?id=com.superevent.hopesummit |
| 1166 | الطب البديل والطب النبوي القديم علاج الاعشاب 2018  /store/apps/details?id=com.app.best.ever.perfectbody |
| 1170 | EonCalculator  /store/apps/details?id=a1byte.co.lungdirect_android |
| 1171 | airbubbl  /store/apps/details?id=com.r4s.airbubbl |
| 1179 | Sigaraya Son  /store/apps/details?id=com.mobincube.sigaraya_son.sc_5WYR1C |
| 1181 | آزمایشگاه همراه  /store/apps/details?id=eng.maria.amani.lab2 |
| 1182 | Brain Tumor: Causes, Diagnosis, and Treatment  /store/apps/details?id=com.healthinfo.braintumor.cause.prevention.management.treatment.diagnosis |
| 1190 | YOU CAN QUIT SMOKING  /store/apps/details?id=com.medfree.mlc |
| 1191 | Chest X-Ray Interpretation  /store/apps/details?id=com.andromo.dev712667.app794545 |
| 1193 | Medicina natural gratis, plantas que curan  /store/apps/details?id=com.victormartinezb.medicinanatural |
| 1194 | সিগারেট খেলে যে সব ক্ষতি হয় ~ Harm of cigarette  /store/apps/details?id=com.Medical.Tobacco.Causes.Treatment |
| 1200 | Pleurisy Disease  /store/apps/details?id=com.bedieman.pleurisyDisease |
| 1201 | Cancer and Tumors  /store/apps/details?id=com.referencehunt.cancerandtumors |
| 1203 | Home Remedies For Cough  /store/apps/details?id=com.healthcareit.Home.Remedies.For.Cough |
| 1207 | Plantas Medicinales y Medicina Natural  /store/apps/details?id=com.ginortcar.plantas.medicinales.medicina.natural |
| 1208 | 표적치료연구회  /store/apps/details?id=kr.procuratio.android.kastt |
| 1210 | the fault in our stars  /store/apps/details?id=com.love.stars |
| 1211 | The Basics Of Chest X-Ray Interpretation  /store/apps/details?id=com.radiology.chest.xray.interpretation |
| 1213 | Common Causes Of Cervical Cancer  /store/apps/details?id=com.andromo.dev673306.app698662 |
| 1219 | Test de Cancer  /store/apps/details?id=com.aldair.jimenez.sena.colomboaleman |
| 1220 | Medical Terminologies Dictionary-Medical Terms  /store/apps/details?id=com.medical.offline.medicaldictionary |
| 1221 | Nutrino - Health Nutrition Facts to Fight Disease  /store/apps/details?id=com.berisco.nutrino |
| 1222 | Wet Cough  /store/apps/details?id=com.andromo.dev565055.app882334 |
| 1232 | Piel Guí­a del Cáncer  /store/apps/details?id=com.andromo.dev522931.app898513 |
| 1236 | Mesothelioma - Asbestos  /store/apps/details?id=com.mesothelioma.asbestosis |
| 1239 | ছোট রোগের ঘরোয়া চিকিৎসা  /store/apps/details?id=com.InnovativeBanglaApps.ChotoRogerGhorowaChikitsa |
| 1240 | Doctor Fruit - Healthy Fruit  /store/apps/details?id=co.doctorfruit.Healthyfruit |
| 1245 | কাঁচা টমেটোর কিছু উপকারিতা  /store/apps/details?id=com.itjogot.useoftomato |
| 1248 | Doctor Vegetable - Healthy Veg  /store/apps/details?id=healthyvegetables.doctorvegetable |

## List of mHealth Apps included in the case study

| 13 | BECCA - Breast Cancer Support  /store/apps/details?id=com.yourcompany.becca |
| --- | --- |
| 16 | EmotionSpace cáncer de mama  /store/apps/details?id=com.pfizer.es.EmotionSpace |
| 19 | chemoWave: for cancer patients  /store/apps/details?id=com.chemowave.android |
| 28 | OWise breast cancer  /store/apps/details?id=nl.onesixty.owise |
| 29 | My Cancer Coach  /store/apps/details?id=com.genomichealth.mycancercoach |
| 34 | Breast Advocate  /store/apps/details?id=com.breastadvocate.android |
| 35 | Breast Cancer Support  /store/apps/details?id=com.myhealthteams.MyBCTeam |
| 36 | KMBCN  /store/apps/details?id=org.kobura.kmbcn |
| 42 | Triple Negative Breast Cancer  /store/apps/details?id=com.Kognito.TNBC |
| 54 | Breast Cancer - Others Like Me  /store/apps/details?id=com.OthersLikeMe.BreastCancer |
| 88 | Outcomes4Me  /store/apps/details?id=com.outcomes4me |
| 90 | Boobytrapp - The Breast Cancer App  /store/apps/details?id=com.boobytrapp |
| 113 | The BAPS App Wales  /store/apps/details?id=com.velindre.baps |
| 121 | BELONG Beating Cancer Together  /store/apps/details?id=com.belongtail.belong |
| 150 | Diana  /store/apps/details?id=com.roche.diana |
| 195 | Got Boobs?  /store/apps/details?id=com.app.gotboobs |
| 242 | inKind Space  /store/apps/details?id=io.pixeledge.inkind |
| 250 | Cancer Surveillance  /store/apps/details?id=com.gomlv.cancersurveillance |
| 300 | Focalyx  /store/apps/details?id=com.teravision.focalyx.android |
| 317 | Adrenal Cancer - Others Like Me.  /store/apps/details?id=com.SameAsIAcc |
| 319 | How Are You Today? PC  /store/apps/details?id=com.intelesant.pc |
| 375 | Cancer.Net Mobile  /store/apps/details?id=com.fueled.cancernet |
| 401 | TNM Cancer Staging  /store/apps/details?id=com.app.tnm |
| 476 | Untire: Beating cancer fatigue  /store/apps/details?id=com.tiredofcancer |
| 511 | Self-Care During Cancer  /store/apps/details?id=com.nearspace.selfcare |
| 557 | CanDi - Cancer Diet App  /store/apps/details?id=com.brok3n.candiapps.candiapps |
| 595 | CancerAid  /store/apps/details?id=au.com.canceraid |
| 628 | GRYT Health Cancer Community  /store/apps/details?id=com.grytapp |
| 696 | Target Ovarian Cancer Symptoms Diary  /store/apps/details?id=uk.org.targetovariancancer.symptomdiary |
| 731 | Pancreatic Cancer Action - symptom tracker  /store/apps/details?id=com.healthbit.pca |
| 733 | My Care Plan (cancer survivor)  /store/apps/details?id=com.nearspace.mycareplan |
